# NS5-independent Ablation of STAT2 by Zika virus to antagonize interferon signalling

**DOI:** 10.1080/22221751.2021.1964384

**Published:** 2021-08-15

**Authors:** Jun Shu, Xiao Ma, Yang Zhang, Jingyi Zou, Zhenghong Yuan, Zhigang Yi

**Affiliations:** a Key Laboratory of Medical Molecular Virology (MOE/NHC/CAMS), School of Basic Medical Sciences, and Shanghai Institute of Infectious Disease and Biosecurity, Fudan University, Shanghai, People’s Republic of China; b Shanghai Public Health Clinical Center, Fudan University, Shanghai, People’s Republic of China

**Keywords:** Flavivirus, Zika virus (ZIKV), interferon, interferon antagonize, STAT2, *de novo* protein synthesis

## Abstract

*Flavivirus* genus includes numerous arthropod-borne human pathogens that are clinically important. Flaviviruses are notorious for their ability to antagonize host interferon (IFN) induced anti-viral signalling. It has been documented that NS5s of flaviviruses mediate proteasome degradation of STAT2 to evade IFN signalling. Deciphering the molecular mechanism of the IFN antagonism by the viruses and reversing this antagonism may dictate anti-viral responses and provide novel antiviral approaches. In this report, by using Zika virus (ZIKV) as a model, we first demonstrated that ZIKV antagonized interferon signalling in an infectious dose-dependent manner; in other words, the virus antagonized interferon signalling at a high multiple of infection (MOI) and was sensitive to interferon signalling at a low MOI. Mechanistically, we found that ZIKV infection triggered degradation of ubiquitinated STAT2 and host short-lived proteins while didn't affect the proteasome activity *per se*. ZIKV infection resulted in suppression of host *de novo* protein synthesis. Overexpression of NS5 alone only marginally reduced STAT2 and had no effect on the host *de novo* protein synthesis. Ectopically expressed murine STAT2 that was resistant to NS5- and ZIKV-induced ablation exaggerated the IFN-induced anti-viral signalling. These data favour a new model of the innate immune evasion of ZIKV in which the viral infection triggers suppression of host *de novo* protein synthesis to accelerate the degradation of short-lived, ubiquitinated STAT2. As flaviviruses share a very conserved replication strategy, the mechanisms of IFN antagonism elucidated here might also be employed by other flaviviruses.

## Introduction

Members of the *flavivirus* genus, such as dengue virus (DENV), West Nile virus (WNV), Japanese encephalitis virus (JEV), tick-borne encephalitis virus (TBEV) and the recently re-emerged Zika virus (ZIKV), infect humans worldwide. ZIKV was first isolated in 1947 [1,2] and then re-emerged in the 1980s in Southeast Asia and in 2007 in Micronesia. Recently, ZIKV has spread in the Americas since 2014 [3]. ZIKV infection causes Guillain-Barre syndrome and microcephaly [4–7]. There have been local ZIKV infections in approximately 87 countries and regions around the world [8]. Recently, African-lineage ZIKV was detected in Brazil for the first time, warning that a new pandemic may occur [9]. Currently, there is no specific vaccine or treatment available for ZIKV infection.

Similar to other flaviviruses, the 10.7-kb positive-sense RNA genome of ZIKV encodes a single open reading frame that is translated into a polypeptide. The polyprotein is co- and posttranslationally processed by host proteinases or the viral protease to produce the viral structural proteins C, prM/M and E and nonstructural proteins NS1, NS2A, NS2B, NS3, NS4A, NS4B, and NS5 [10]. ZIKV has broad cell tropism, infecting various cells, including human skin cells, myeloid cells and human progenitor cells of neuronal, placental and testicular origin. While ZIKV infection induces strong interferon production in certain cell types, such as A549 cells [11,12], it has been demonstrated type I IFNs inhibits ZIKV replication in several human cell types and in mouse models. Type I IFN binds to interferon receptors (IFNAR1 and IFNAR2) on the cell surface and activates the Janus kinases Jak1 and Tyk2, which in turn recruit the transcription signal transducers STAT1 and STAT2 and phosphorylate STAT1 and STAT2 [13]. Phosphorylated STAT1 and STAT2 form a heterodimer and engage IFN regulatory factor 9 (IRF-9) to assemble interferon-stimulating gene factor 3 (ISGF3) [14,15]. ISGF3 translocates into the nucleus and binds to IFN-sensitive response elements (ISREs) located in the upstream promoter regions of interferon-stimulating genes [16], resulting in upregulation of IFN-stimulating genes (ISGs) to exert antiviral activity [17].

Similar to other flaviviruses, ZIKVs are also notorious for their ability to evade Type I IFN responses by diverse strategies [18–21]. A multitude of viral nonstructural proteins have been involved in inhibition of the signalling pathways that lead to type I interferon induction and IFN-mediated expression of IFN-stimulated genes (ISGs). Recent studies have uncovered some of the mechanisms by which ZIKV and other flaviviruses inhibit IFN signalling. DENV and ZIKV infection induces degradation of STAT2 [22–24]. DENV NS5 induces degradation of STAT2 in a proteasome-dependent manner through the E3 ubiquitin ligase UBR4 [24]. Flaviviruses also interrupt the phosphorylation of IFN signalling components. DENV NS4B inhibits the phosphorylation and nuclear translocation of STAT1 [25]; JEV NS5 inhibits Tyk2 and STAT1 tyrosine phosphorylation and STAT1 nuclear translocation [26]; NS4A of TBEV can block the phosphorylation of STAT1 and STAT2 and the dimerization of STAT1/STAT2 [27]; ZIKV reduces the phosphorylation of STAT1 [12,28]; and WNV blocks the phosphorylation and activation of Jak1 and Tyk2 [29]. Meanwhile, NS5 of TBEV and WNV inhibit the cell surface expression of IFNAR1 [30]. NS2B-NS3 of ZIKV degrades Jak1 [31]. Taken together, these studies support the general concept that ZIKV works effectively to inhibit IFNAR signalling in many different cell types and NS5 plays a crucial role in IFN antagonism. However, the detailed molecular mechanisms have not been elucidated.

In this study, we generated infectious clones of ZIKV MR766 (C7) and a mutant with a 29-nt deletion in the 3′-NTR (C7.D29), which presumably renders the virus sensitive to IFN, as reported in DENV [32–34]. Using the C7 and C7.D29 viruses as models, we found that ZIKV antagonized interferon signalling in an infection dose-dependent manner and that the virus antagonized interferon signalling at a high multiple of infection (MOI) and was sensitive to interferon signalling at a low MOI. Mechanistically, ZIKV infection accelerated the turnover of ubiquitinated host proteins and STAT2, probably by interrupting host *de novo* protein synthesis. Conversly, overexpression of NS5 alone barely contributed to this process. Furthermore, overexpression of murine STAT2 that was refractory to ZIKV-induced degradation elicited robust antiviral signalling. Hence, we uncovered an NS5-indepenent pathway utilized by ZIKV to evade IFN signalling.

## Materials and methods

### Plasmids

To generate the plasmid pLenti-puro-ISRE-HA-RFP, the cassette of ISRE-minP in the pGL4.45 [luc2P ISRE Hygro](Promega) was first PCR amplified and cloned into the SanDI/NsiI site in the Lentipuro-guide plasmid (addgene, 52963) to get a temporal plasmid. And then the HA-RFP-hPEST cassette was cloned into the KpnI/BamHI site in the temporal plasmid to get the final plasmid pLenti-puro-ISRE-HA-RFP. To generate the plasmid pTrip-IRES-puro-GFPu, GFPu was amplified from the plasmid TCRα-GFPu (kindly gifted by professor Ron Kopito) and cloned into the XbaI/BamHI site in the plasmid pTrip-IRES-puro. To generate the plasmid pTrip-IRES-BSD-HA-Ub, HA-Ub cassette was cloned into the XbaI/XhoI site in a homemade plasmid pTrip-IRES-BSD. To generate the plasmid pTrip-IRES-puro-GFP, GFP coding sequence was cloned into the XbaI/BamHI site in a homemade plasmid pTrip-IRES-puro. To Generate the plasmids pTRIP-IRES-puro-mSTAT2-HA, coding regions of STAT2 were amplified from the cDNA clones (Mouse Tagged ORF Clone MR227437 and MR225472, OriGene), flanking with the HA tag in the C-termini of the ORFs, and then cloned into the XhoI/BamHI site in the plasmid pTrip-IRES-puro, respectively. To generate the plasmid pTRIP-IRES-puro-STAT2-HA, coding sequence for human STAT2 and a C-terminally flanked HA tag was synthesized and cloned into the BsrGI/XhoI site in the plasmid pTRIP-IRES-puro. To generate plasmid phCMV-Ub-NS5, ubiquitin gene was fused to the N-terminal of ZIKV NS5 by fusion PCR and then the fused genes were cloned into the BglII/EcorI site in phCMV (Genlantis). The suppressor tRNA plasmid (pSVB.Yam) and mutant amino-acyl tRNA synthetase plasmid for *p*-azido-L-phenylalanine (pcDNA.RS) [35] were kindly provided by professor Thomas P. Sakmar (Rockefeller University).

To generate the infectious clones of Zika virus, five DNA fragments encompassing the whole Zika virus MR766 genome (AY632535.2) were synthesized (Genewiz, Suzhou), assembled and cloned stepwise into pACNR vector [36] (kindly gifted by professor Charles Rice). The clones were corrected according to another version of MR766 genome sequence (KU955594.1) by fusion PCR-mediated mutagenesis. After seven rounds of mutation, the finalized clone was named as C7. A 29nt region in the 3′-NTR was deleted in the C7 to get the plasmid C7.D29. *Gaussia* luciferase (Gluc) was inserted in the N-terminal of the viral ORF similarly as reported [37] to get the plasmids C7-Gluc and C7.D29-Gluc.

### Cell lines

HEK293T cells, Vero cells (Cell Bank of the Chinese Academy of Sciences, Shanghai, China) and Huh7.5 (kindly provided by Charles Rice) were routinely maintained in Dulbecco modified medium supplemented with 10% fetal bovine serum (Biological Industries catalog no. 04-001-1), 25 mM HEPES (Gibco), and nonessential amino acids (Gibco). Human choriocarcinoma JEG-3 cell line (Cell Bank of the Chinese Academy of Sciences, Shanghai, China) were maintained in Minimum Essential Medium supplemented with 10% fetal bovine serum (Gibco catalog no. 10099-141C), 25 mM HEPES (Gibco), and Sodium Pyruvate (Gibco). Glioblastoma SF268 cell line (Shanghai Baiye Biotechnology Center Shanghai, China) were maintained in Dulbecco modified medium supplemented with 10% fetal bovine serum (Gibco catalog no. 10099-141C) and 25 mM HEPES (Gibco). Huh7.5 cells harbouring pTrip-IRES-puro-ISRE-HA-RFP, pTrip-IRES-puro-GFPu, pTrip-IRES-BSD-HA-Ub, pTrip-IRES-puro-GFP, pTrip-IRES-puro-hSTAT2, pTrip-IRES-puro-mSTAT2-HA were generated by transducing Huh7.5 cells with vesicular stomatitis virus G protein (VSV-G)-pseudotyped lentiviral particles and growing cells in conditioned medium supplemented with 5 μg/ml puromicin or blasticidin, respectively. The surviving cells were pooled and maintained in conditioned medium with 0.5 μg/ml puromicin or blasticidin.

### Antibodies and chemicals

An anti-β-Actin antibody (A1978; Sigma) was used for Western blotting at a 1:4,000 dilution. Anti-HA antibody from Roche (11867423001) was used in Western blotting analyses at a 1:500 dilution. Anti-GFP antibody from Santa Cruz Biotechnology (sc-9996) was utilized at a 1:1,000 dilution for Western blotting. Anti-p53 (a rabbit polyclonal antibody, SantaCruz Biotechnology, SC-6243) was used for Western blotting at a 1:350 dilution. Rabbit anti-STAT2 antibody (sc-476; Santa Cruz) was used at a 1:1,000 dilution for Western blotting. Anti-PARP (A19596; ABclonal) was used at a 1:1000 dilution for Western blotting. Anti-ZIKV NS5 antibody (GTX133312; Genetex) and anti-ZIKV NS3 antibody (GTX133309; Genetex) were used in Western blots at 1:1000 dilution and 1:500 dilution in immunostaining. Anti-YFV NS5 antibody (GTX134141; Genetex) were used in Western blots at 1:1000 dilution in immunostaining. Goat-anti-mouse HRP IgG (Santa Cruz; sc-2005) was used at 1:2000 dilution; goat-anti-rat HRP IgG (Santa Cruz; sc-2006) was used at 1:2000 dilution; goat-anti-rabbit HRP IgG (Santa Cruz; sc-2004) was used at 1:2000 dilution; Alexa Fluor 488 goat-anti-rabbi IgG (Life technologies; A11008) was used at 1:500 dilution; goat-anti-mouse IRDye 800CW secondary antibody (licor; 926-32210) was used at 1:10000 dilution; goat-anti-rabbit IRDye 800 CW secondary antibody (licor; 926-32211) was used at 1:10000 dilution.

IFN-α was purchased from pbl Assay Science (11200-2). MG132 (M7449; Sigma) was used at a final concentration of 10 μM. 4-Azido-L-phenylalanine (33173-53-4; MCE) was used at a final concentration of 0.5 mM. Polybrene was purchased from Santa Cruz Biotechnology (sc-134220). Leu-Leu-Val-Tyr-AMC was purchased from Enzo Life Sciences (Farmingdale, NY).

### Virus

The ZIKV plasmids were linearized by AfeI (Fermentas, fast digestion) digestion and purified by MinElute Gel Extraction Kit (Qiagen). DNAs were eluted in nuclease-free water and used as templates in *in vitro* transcription by mMESSAGE mMACHINE kit (Ambion). The *in vitro*-transcribed RNAs were purified by RNeasy Mini Kit (Qiagen) and eluted in nuclease-free water. ZIKV was generated by electroporation of *in vitro*-transcribed viral RNAs into Vero cells, and the virus titer was determined in Vero cells by plaque assay. The VSV stock was obtained from the Shanghai Public Health Clinical Center. YFV (strain 17D) stocks were generated by transfection with in vitro-transcribed RNA from plasmid pACNRYF17D as previously described [38] and titers were determined in Vero cells by plaque assay. VSV-G-pseudotyped lentiviral particles were generated by co-transfection of HEK293T cells with plasmids encoding VSV-G and HIV gag-pol and with the lentiviral provirus plasmids. The medium overlying the cells was harvested at 48–72 h after transfection, filtered through a 0.45 μm filter, and stored at −80°C. Cells were transduced with the pseudoparticles in the presence of 8 μg/ml polybrene.

### Plaque assay and TCID_50_

ZIKV, VSV and YFV were titered by infection of Vero cells with 10-fold serial dilutions in DMEM with 2% FCS. 200 μl of diluted virus was added to each well in 6-well plate and after 1 h of infection the well was overlaid with 0.6% agarose in MEM supplemented with 2% FBS. Plaques were enumerated by crystal violet staining after 3 days for C7 and 6 or 7 days for ZIKV C7-Gluc and 36 h for VSV.

As the C7.D29-Gluc did not form plaque in Vero cells, we determined the titer by measuring the TCID_50_. C7-Gluc and C7.D29-Gluc were 10-fold serial diluted as described above and then viruses in each dilution infected 8 wells of Vero cells in 96-well plates. After 5 days post infection, the cells were fixed and stained with anti-ZIKV NS3 antibody followed by Alex488-conjugated secondary antibody and observed by fluoresce microscopy. For each virus dilution, the numbers of NS3-postive wells were counted and the virus titer was calculated by Reed_Muench method.

### Luciferase activity

Cells in 48-well plate were lysed in 60 μl of 1 × passive lysis buffer (Promega). A 10 μl of lysate was mixed with 50 μl Renilla luciferase substrate (Promega) and the luciferase activity was measured by a GLOMX luminometer (Promega).

### Quantitative reverse transcription (RT)-PCR

RNAs were extracted using the TRIzol reagent and reverse transcribed with the PrimeScript RT reagent kit with gDNA Eraser from TaKaRa according to the manufacturer's instructions. The cDNA samples were subjected to real-time PCR (SYBR Premix Ex Taq Tli RNase H Plus) with the following primers for specific genes: GAPDH, (s [sense] GGT ATC GTG GAA GGA CTC ATG A) and (as [antisense] ATG CCA GTG GCT TCC CGT TCA GC); ZIKV, (s [sense] GGC GGT CAG TGG AGA TGA CTG C) and (as [antisense] CCG GAT GCT CCA TCC TGC C); VSV, (s) GAT AGT ACC GGA GGA TTG ACG ACT A and (as) TCA AAC CAT CCG AGC CAT TC. The relative RNA levels were calculated by the 2^−ΔΔCT^ method [39]. The gene for glyceraldehyde 3-phosphate dehydrogenase (GAPDH) was used as a housekeeping gene for loading normalization.

### Proteasome activity assays

The detection method of proteasome activity is based on the principle of detecting fluorophore 7-amino-4 metholcoumarin (AMC) cleaved by 20S proteasome from the labelled substrate Leu-Leu-Val-Tyr-AMC [40]. Cells were washed with PBS for three times, and then lysed with analytical lysis buffer (50 mM Tris-HCl, pH 7.5; 150 mM NaCl; 1% Triton X-100; 2 mM ATP). The cell lysate was placed on ice for 30 min, vortex every 5 min, and then centrifuged at 12000 g for 10 min. The supernatant after centrifugation was collected and the protein concentration was determined by BCA kit. Ten microgram of cell proteins was diluted to 90 μl with analytical buffer (50 mM Tris HCl, pH 7.5; 150 mM NaCl). Then 10 μl of the synthetic fluorogenic substrates Suc-Leu-Leu-Val-Tyr-AMC (500 μM, Enzo Life Sciences) was added to each assay reaction (final concentration of 50 μM in a total volume of 100 μl). The samples were incubated at 37°C and the fluorescence signal was read in Molecular Devices (Flexstation 3) microplate detection system with excitation wavelength of 355 nm and emission wavelength of 460 nm.

### Western blotting

After washing with PBS, cells were lysed with 2 × SDS loading buffer [100 mM Tris-Cl (pH 6.8), 4% SDS, 0.2% bromophenol blue, 20% glycerol, 10% 2-mercaptoethanol] and then boiled for 10 min. Proteins were separated by SDS PAGE and transferred to a nitrocellulose membrane. The membranes were incubated with blocking buffer (PBS, 5% milk, 0.05% Tween) for 1 h and then with primary antibody diluted in the blocking buffer. After three washes with PBST (PBS, 0.05% Tween), the membranes were incubated with secondary antibody. After three washes with PBST, the membrane was visualized by Western Lightning Plus-ECL substrate (PerkinElmer; NEL10500) or by an Odyssey CLx Imaging System. The protein bands were quantified by densitometry with ImageJ if necessary.

### Immunoprecipitation

Cells in six-well plates were lysed with 300 µl lysis buffer [50 mM TrisCl (pH 7.5), 1 mM EDTA, 0.5% SDS, 200 mM NaCl, 20 µg/ml phenylmethylsulfonyl fluoride, proteinase inhibitor (Roche)]. Cell lysates were then passed through a 27-gauge needle 10 times. A 30 µl aliquot of the supernatant was taken and mixed with an equal volume of 2 × SDS loading buffer as input (10 %). The remaining clarified cell lysates were incubated for 4 h at 70°C and then add 300 µl 2 × TNA (0.25% TritonX100, 1 mg/ml BSA, 50 mM Tris-Cl, 200 mM NaCl, 1 mM EDTA, 20 µg/ml phenylmethylsulfonyl fluoride), and then add 600 µl 1×TNA and centrifuged at 12,000 g for 10 min. After centrifugation, the supernatant was incubated with 20 µl anti-HA magnetic beads (Pierce; SB246262) with rotation at 4°C overnight. After four washes with 1× TNA, the beads were lysed with 2×SDS loading buffer. The samples were boiled for 10 min then analysed by western blotting.

### Statistical analysis

Statistical analysis was performed with the GraphPad Prism 6 software. Specific tests are described in the figure legends.

## Results

### Multiplicity of infection (MOI)-dependent sensitivity of ZIKV to interferon-α (IFN-α)

To elucidate the molecular mechanism of IFN antagonism by ZIKV, we first generated an infectious clone of ZIKV MR766. Five DNA fragments encompassing the whole genome of Zika virus MR766 (KU955594.1) were synthesized and assembled to form a full-length cDNA clone (C7). We also generated mutant C7.D29 (Supplementary Figure 1a) with a 29-nt deletion in the 3′-NTR, similar to DENV, which presumably renders viruses sensitive to IFN, as reported in DENV [32–34]. A similar deletion in the ZIKV Cambodian strain FSS13025 resulted in a mutated strain that was more sensitive to IFN than the wild-type virus [41]. When the *in vitro*-transcribed C7 and C7.D29 RNAs were transfected into Vero cells, viruses generated from C7 and C7.D29 were released following similar kinetics, whereas the C7.D29 virus showed less cytopathic (Supplementary Figure 1b) and formed plaques with smaller sizes (Supplementary Figure 1c). C7.D29 exhibited slower replication kinetics in Huh7.5 cells, as much fewer NS3 and NS5 proteins (Supplementary Figure 1d) and significantly lower viral RNA levels (Supplementary Figure 1e) in C7.D29-infected cells than in the C7-infected cells. To facilitate detection, we also generated cDNA clones (C7-Gluc and C7.D29-Gluc) with reporter gene *Gaussia* luciferase (Gluc) into the viral genome according to the strategy used for other flaviviruses [37]. The C7-Gluc formed plaques in Vero cells with smaller size than C7 (Supplementary Figure 1f) without losing virus titer (data not shown). The C7.D29-Gluc could not form plaques in Vero cells (data no shown).

**Figure 1.**
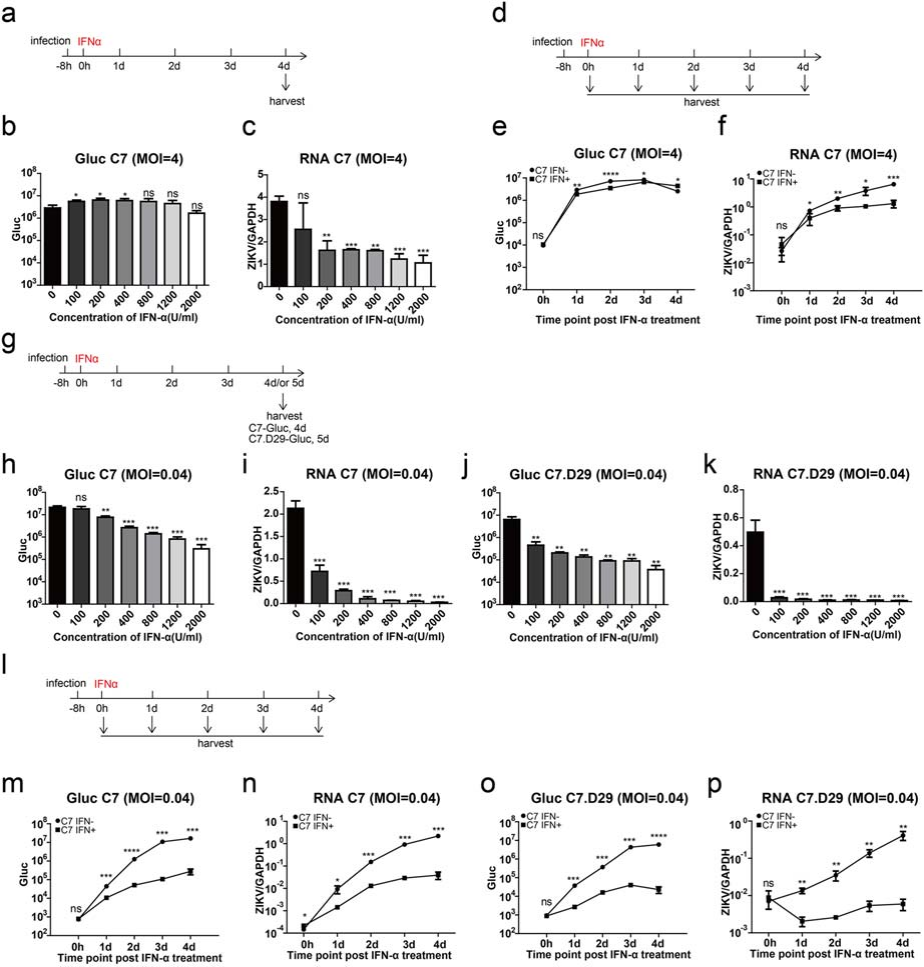
Multiplicity of infection (MOI)-dependent sensitivity of ZIKV to interferon-α (IFN-α). (a) Experimental design for (b) and (c). Huh7.5 cells were infected with C7-Gluc (MOI = 4) for 8 h and then treated with various concentrations of IFN-α. Cells were harvested at 4 d post infection. (b) Gaussia luciferase (Gluc) activity in the cell lysates was determined. The mean ± SD of three biological replicates is shown (*n* = 3). (c) ZIKV RNA from infected cells was quantified by RT-PCR and normalized to the level of GAPDH RNA. The mean ± SD of three biological replicates is shown (*n* = 3). (d) Experimental design for (e) and (f). Huh7.5 cells were infected with C7-Gluc (MOI = 4) for 8 h and then treated with IFN-α (2000 U/ml). Cells were harvested at the indicated time points after IFN-α treatment. (e) Gluc activity in the cell lysates was determined. The mean ± SD of three biological replicates is shown (*n* = 3). (f) ZIKV RNA from infected cells was quantified by RT-PCR and normalized to the level of GAPDH RNA. The mean ± SD of three biological replicates is shown (*n* = 3). (g) Experimental design for (h–k). Huh7.5 cells were infected with C7-Gluc or C7.D29-Gluc (MOI = 0.04) for 8 h and then treated with various concentrations of IFN-α. C7-Gluc-infected cells were harvested at 4 d post infection, and C7.D29-Gluc-infected cells were harvested at 5 d post infection. (h, j) Gluc activity in the cell lysates was determined. The mean ± SD of three biological replicates is shown (*n* = 3). (i, k) ZIKV RNA from infected cells was quantified by RT-PCR and normalized to the level of GAPDH RNA. The mean ± SD of three biological replicates is shown (*n* = 3). (l) Experimental design for (M-P). Huh7.5 cells were infected with C7-Gluc or C7.D29-Gluc (MOI = 0.04) for 8 h and then treated with IFN-α (2000 U/ml). At the indicated time points after IFN-α treatment, cells were harvested. (m, o) The Gluc activity in the cell lysates was determined. The mean ± SD of three biological replicates is shown (*n* = 3). (n, p) ZIKV RNA from infected cells was quantified by RT-PCR and normalized to the level of GAPDH RNA. The mean ± SD of three biological replicates is shown (*n* = 3). Statistical analysis was performed between IFN-treated groups and untreated groups (ns, not significant, *P < 0.05, **P < 0.01, ***P < 0.001; two-tailed, unpaired t-test).

To specifically study the effect of ZIKV on IFN-triggered signalling pathways, we chose Huh7.5 cell, which is deficient in IFN induction pathways [42,43]. We infected Huh7.5 cells with C7-Gluc at a MOI of 4 and then treated the cells with various concentrations of IFN-α at 8 h post infection (Figure 1(a)). At 4 d post infection, viral replication of C7-Gluc was only moderately reduced by IFN-α treatment, as assessed by measuring Gluc activity (Figure 1(b)) and viral RNA levels (Figure 1(c)). Then, we infected Huh7.5 cells with C7-Gluc at an MOI of 4 for 8 h and treated them with 2000 U/ml IFN-α. Viral replication was assessed at various time points after treatment (Figure 1(d)). Similarly, IFN-α treatment significantly but only moderately reduced Gluc activity (Figure 1(e)) and viral RNA levels (Figure 1(f)). We also tested the antiviral activity of IFN-α against ZIKV when IFN-α was administered prior to virus infection. We pretreated Huh7.5 cells with 2000 U/ml IFN-α 8 h before C7-Gluc infection (MOI, 4). IFN-α dramatically reduced Gluc activity at early time points but only moderately reduced Gluc activity at late time points post infection (Supplementary Figure 2a-b).

We then attempted to compare C7-Gluc and C7.D29-Gluc. The titer of C7.D29-Gluc was relatively low, and we infected Huh7.5 cells with C7-Gluc or C7.D29-Gluc at an MOI of 0.04. At 8 h post infection, the infected cells were treated with various concentrations of IFN-α and then harvested 4 d (for C7-Gluc) or 5 d (for C7.D29-Gluc) post infection (Figure 1(g)). In contrast to infection with a high MOI, under these conditions, both C7-Gluc and C7.D29-Gluc were sensitive to IFN-α treatment in a dose-dependent manner, as evidenced by dramatic reductions in Gluc activity and viral RNA levels in IFN-treated cells (Figure 1(h–k)). Notably, C7.D29-Gluc was more sensitive to IFN treatment compared to C7-Gluc when treated with IFN at a concentration of 100 U/ml, as evidenced by Gluc activity and viral RNA levels (Figure 1(h–k)). When the C7-Gluc- and C7.D29-Gluc-infected cells were treated with 2000 U/ml IFN-α and harvested at various time points after treatment (Figure 1(l)), the Gluc activity and viral RNA levels in both C7-Gluc- and C7.D29-Gluc-infected cells were dramatically reduced compared with untreated cells (Figure 1(m–p)).

We also verified if the sensitivity of virus without Gluc to IFN is consistent with recombinant ZIKV with Gluc, Huh7.5 cells were infected with C7 and C7.D29 under the same conditions. At 8 h post infection, the infected cells were treated with various concentrations of IFN-α and then harvested 4 d post infection. The results showed that the viral RNA levels in both C7 and C7.D29 infected cells were dramatically reduced compared with untreated cells (Supplementary Figure 2c-e), which is consistent with recombinant ZIKV with Gluc.

Taken together, these data suggest that the IFN sensitivity of ZIKV depends on the MOI used for infection and that C7.D29 is more sensitive to IFN-α treatment than is C7.

### ZIKV actively antagonized interferon signalling

**Figure 2.**
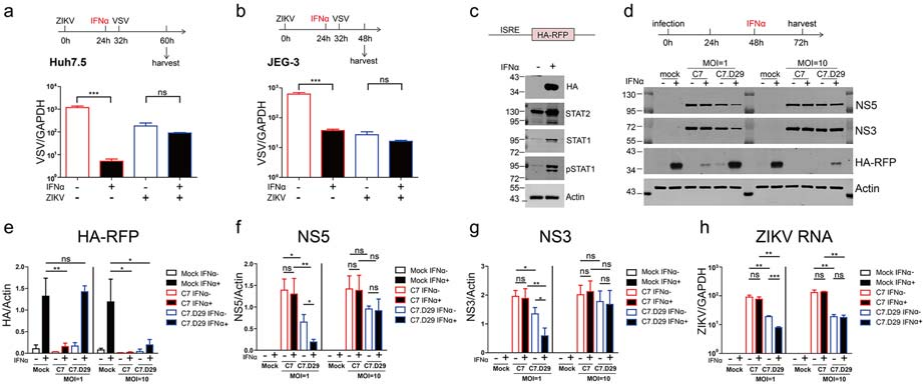
ZIKV actively antagonized interferon signalling. Coinfection with ZIKV abrogated the IFN-induced reduction in VSV infection in Huh7.5 cells (a) and JEG-3 cells (b). (a) Huh7.5 cells were infected with ZIKV (MOI = 1) for 24 h and then treated with IFN-α (2000 U/ml) for 8 h, followed by infection with VSV (MOI = 0.05) for 28 h. The levels of VSV genomic RNA in the harvested cells were determined by qRT-PCR and normalized to the level of GAPDH RNA. The mean ± SD of three biological replicates is shown (*n* = 3). (b) JEG-3 cells were infected with ZIKV (MOI = 1) for 24 h and then treated with IFN-α (2000 U/ml) for 8 h, followed by infection with VSV (MOI = 0.05) for 16 h. The levels of VSV genomic RNA in the harvested cells were determined by qRT-PCR and normalized to the level of GAPDH RNA. The mean ± SD of three biological replicates is shown (*n* = 3). (c) western blotting analysis of the Huh7.5 cell line expressing the ISRE-HA-RFP reporter. Cells were treated (+) or not (−) with IFN-α (2000 U/ml) for 24 h and then analysed by western blotting with the indicated antibodies. A representative picture of multiple independent experiments is shown. (d–h) ZIKV antagonized ISRE-dependent IFN signalling. (d) The upper panel shows the experimental design for c–f. Huh7.5-ISRE-HA-RFP cells were infected (+) or not (−) with C7 or C7.D29 at an MOI of 1 or 10 for 48 h and then treated (+) or not (−) with IFN-α (2000 U/ml) for 24 h. The harvested cells were analysed by western blotting with the indicated antibodies. Representative blots of three biological replicates are shown. The values to the left of the blots are molecular sizes in kilodaltons. (e–g) The protein abundances of each protein in C were quantified and plotted. The mean ± SD of three biological replicates is shown (*n* = 3). (h) ZIKV RNA levels were quantified by quantitative RT-PCR and normalized against GAPDH RNA levels. The mean ± SD of three biological replicates is shown (*n* = 3). Statistical analysis was performed between C7- or C7.D29-infected groups and uninfected groups (ns, not significant, *P < 0.05, **P < 0.01, ***P < 0.001; two-tailed, unpaired t-test).

To address whether ZIKV actively suppresses IFN signalling, we monitored the antiviral activity of IFN against VSV, which is a virus that is very sensitive to IFN, in ZIKV-infected cells. We first infected Huh7.5 cells with ZIKV at an MOI of 1 for 24 h and then treated the cells with 2000 U/ml IFN-α. Eight hours later, we infected the cells with VSV and determined the viral RNA levels of VSV after another 28 h (Figure 2(a)). In ZIKV-uninfected cells, VSV was sensitive to IFN-α treatment, as the viral RNA level was dramatically reduced upon IFN-α treatment. In contrast, in ZIKV-infected cells, VSV was refractory to IFN-α treatment, and no significant reduction in viral RNA levels was observed (Figure 2(a)), suggesting active suppression of IFN signalling in ZIKV-infected cells.

To examine if this phenomenon exists in other physiologically related cell line, we repeated the experiments in human choriocarcinoma JEG-3 cell line. We infected JEG-3 cells with ZIKV at an MOI of 1 for 24 h and then treated the cells with 2000 U/ml IFN-α. Eight hours later, we infected the cells with VSV and determined the viral RNA levels of VSV after another 16 h (Figure 2(b)). The results showed that VSV was sensitive to IFN-α treatment in the ZIKV-uninfected cells but refractory to IFN-α treatment, in ZIKV-infected cells, which is consistent with the phenomenon in Huh7.5 cells.

Furthermore, we generated a Huh7.5 stable cell line that expresses HA-RFP driven by IFN-stimulated response elements (ISRE). Upon IFN-α treatment, HA-RFP was readily induced, along with the induction of STAT1 and STAT2 and the phosphorylation of STAT1 (pSTAT1) in Huh7.5-ISRE-HA-RFP cells (Figure 2(c)), demonstrating that ISRE reporter gene expression correlates with the activation of IFN signalling. Then, we infected the reporter cells with C7 or C7.D29 at MOIs of 1 and 10. At 48 h post infection, we treated the cells with 2000 U/ml IFN-α for 24 h and then analysed the reporter gene expression by western blotting and quantified the viral RNA by quantitative RT–PCR (Figure 2(d–h)). When the cells were infected with virus at an MOI of 1, IFN-induced HA-RFP expression was significantly reduced in C7-infected cells but was not affected in the C7.D29-infected cells compared to that in the mock-infected cells (Figure 2(d,e)). Upon IFN-α treatment, NS5 and NS3 protein levels and viral RNA levels were reduced in C7.D29-infected cells, whereas those in C7-infected cells were unaffected (Figure 2(d,f,g)), suggesting IFN antagonism in C7-infected cells but not in C7.D29-infected cells. However, when the cells were infected at an MOI of 10, IFN-induced HA-RFP expression was dramatically reduced in both C7-infeced cells and C7.D29-infected cells (Figure 2(d,e)), and the viral protein levels and RNA levels in C7- and C7.D29-infected cells were unaffected by IFN, indicating that both C7 and C7.D29 antagonize IFN signalling at high MOIs.

Notably, when the cells were infected at a high MOI of 10, IFN-induced HA-RFP expression was reduced in C7.D29-infected cells to a lesser extent than in C7-infected cells (Figure 2(d,e)), suggesting that C7.D29 exhibits less antagonizing activity than C7 does. There were comparable viral protein levels in C7- and C7.D29-infected cells (Figure 2(d,f,g)) but lower viral RNA levels in C7.D29-infected cells (Figure 2(h)). The lower antagonizing activity of C7.D29 is probably due to its lower replication capacity. Taken together, these data indicate that ZIKV actively antagonizes IFN signalling.

### ZIKV infection induced a reduction in STAT2 protein levels

ZIKV infection has been reported to reduce STAT2 expression [12,22,23]. We first examined the effect of ZIKV infection on STAT2. We infected Huh7.5 cells with C7 or C7.D29 at an MOI of 5 and then treated the cells with 400 U/ml IFN-α. We analysed the protein levels STAT2 at 48 and 72 h post infection (Supplementary Figure 3a). At 48 and 72 h post infection, C7 and C7.D29 infection resulted in a significant reduction in STAT2 in the absence or presence of IFN-α treatment, although C7.D29 reduced the protein level of STAT2 to a lesser extent (Supplementary Figure 3b and 3c). IFN-α treatment marginally reduced viral protein levels in C7.D29-infected cells but did not affect viral protein levels in C7-infected cells (Supplementary Figure 3b and 3d). Taken together, these results indicate that ZIKV infection reduces the protein levels of STAT2.

### Zika virus infection accelerated proteasomal degradation

**Figure 3.**
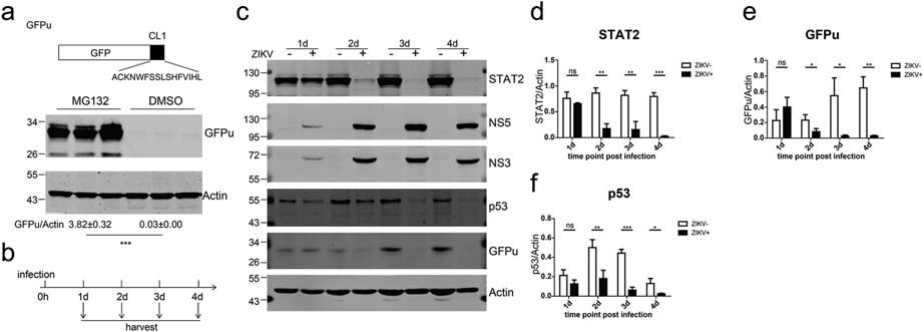
ZIKV infection accelerated proteasomal degradation. (a) Huh7.5 cells stably expressing GFPu (upper panel) in triplicate wells were treated with DMSO or MG132 (10 μM) for 24 h and then analysed by western blotting with the indicated antibodies. (b) Schematic of the experimental design for c–i. Huh7.5-GFPu cells were infected (+) or not (−) with ZIKV C7 (MOI = 1) and harvested at the indicated time points. (c) Western blotting analysis of the cells with the indicated antibodies. Representative pictures of three biological replicates are shown. The values to the left of the blots are molecular sizes in kilodaltons. (d–f) The protein abundances of each protein in B were quantified and plotted. The mean ± SD of three biological replicates is shown (*n* = 3). Statistical analysis was performed between C7-infected groups and uninfected groups (ns, not significant, *P < 0.05, **P < 0.01, ***P < 0.001; two-tailed, unpaired *t*-test).

Previous studies have reported that ZIKV degrades human STAT2 in a proteasome-dependent manner [22,23]. To further investigate this process, we established a reporter system to monitor proteasome activity by expressing an unstable GFP (GFPu) whose carboxyl terminus is fused in frame with a degron (CL1) that is recognized and degraded by the proteasome [44]. When the stable Huh7.5 cell line harbouring GFPu was treated with the proteasome inhibitor MG132, abundant GFPu was detected, indicating efficient inhibition of proteasomal activity by MG132 (Figure 3(a)). We then infected Huh7.5-GFPu cells with ZIKV for various intervals and examined the protein expression of GFPu, STAT2 and the short-lived protein p53, which is a substrate for proteasomal degradation. Starting at 2 d post infection, ZIKV infection resulted in a reduction in STAT2 protein levels (Figure 3(b,d)). Intriguingly, ZIKV infection reduced the expression of GFPu and p53 (Figure 3(c,e,f)).

**Figure 4.**
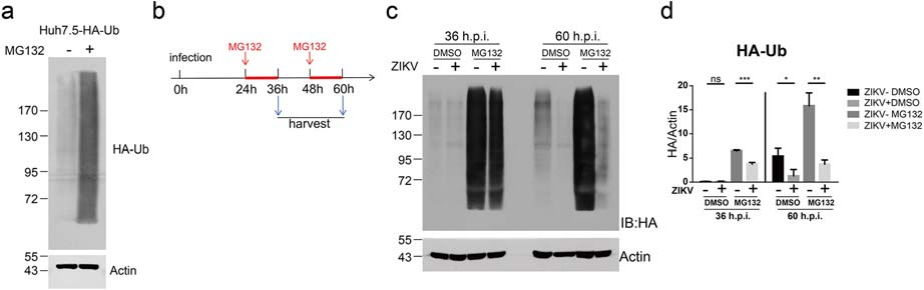
ZIKV infection induced degradation of ubiquitinated proteins. (a) Huh7.5-HA-Ub cells were treated with 10 μM DMSO or MG132 for 12 h and analysed by western blotting with the indicated antibodies. A representative picture of multiple independent experiments is shown. (b) Schematic of the experimental design for C-D. Huh7.5-HA-Ub cells were infected with ZIKV C7 (MOI = 1). At 24 and 48 h post infection, the cells were treated with 10 μM DMSO or MG132 for 12 h and then harvested. (c) Cells were analysed by western blotting with the indicated antibodies. Representative pictures of three biological replicates are shown. The values to the left of the blots are molecular sizes in kilodaltons. (d) The protein abundance of HA-Ub in C was quantified and plotted. The mean ± SD of three biological replicates is shown (*n* = 3). Statistical analysis was performed between C7-infected groups and uninfected groups (ns, not significant, *P < 0.05, **P < 0.01, ***P < 0.001; two-tailed, unpaired *t*-test).

To further explore the effect of ZIKV infection on the proteasome degradation system, we generated a Huh7.5 stable cell line expressing HA-tagged ubiquitin (HA-Ub). This cell line was sensitive to MG132 treatment, resulting in the accumulation of HA-Ub (Figure 4(a)). We infected Huh7.5-HA-Ub cells with C7 for 24 h or 48 h and then treated the infected cells with MG132 for an interval of 12 h. After MG132 treatment, we analysed the ubiquitinated proteins by western blotting with an anti-HA antibody (Figure 4(b)). The MG132-induced accumulation of ubiquitinated proteins was significantly reduced in C7-infected cells at 36 or 60 h post infection (Figure 4(c,d)). Notably, at 60 h post infection, ZIKV infection also significantly reduced the levels of ubiquitinated proteins in the carrier (DMSO)-treated cells (Figure 4(c,d)). These data suggest that ZIKV infection accelerated the proteasomal degradation of ubiquitinated proteins.

We also examined whether ZIKV infection induced degradation of ubiquitinated STAT2. We infected Huh7.5-HA-Ub cells with C7 for 12 h or 24 h and then treated the cells with MG132 for an interval of 12 h. The ubiquitinated proteins were immunoprecipitated by anti-HA beads. Ubiquitinated STAT2 was detected by western blotting with an anti-STAT2 antibody (Supplementary Figure 4a). As described above, STAT2 protein levels were diminished in ZIKV-infected cells and partially restored by MG132 treatment (Supplementary Figure 4b). In the HA co-precipitated samples, ubiquitinated STAT2 can be detected after MG132 treatment (Supplementary Figure 4b). MG132 can basically restore the expression of ubiquitinated STAT2 reduced by ZIKV at 24 h post infection, but only partially restore the expression of ubiquitinated STAT2 reduced by ZIKV at 36 h post infection (Supplementary Figure 4b–d). These results indicated that ZIKV infection reduce the expression of ubiquitinated STAT2. Taken together, these data indicate that ZIKV infection accelerated the proteasomal degradation of host ubiquitinated proteins, including STAT2.

### Proteasome inhibitor MG132 restored ZIKV infection-induced STAT2 degradation at early time points of infection

We sought to restore STAT2 expression in ZIKV-infected cells through the proteasome inhibitor MG132. We infected Huh7.5-GFPu cells with C7 at an MOI of 1. At 2 d post infection, we treated the cells with 10 μM MG132 for 24 h and then analysed STAT2 and GFPu expression (Supplementary Figure 5a). As expected, MG132 treatment resulted in significant accumulation of GFPu proteins in the uninfected cells. Surprisingly, in ZIKV-infected cells, MG132 did not restore STAT2 expression or GFPu expression. We then essentially followed the protocol reported in a previous study, in which MG132 restored ZIKV-induced degradation of STAT2 [23]. We infected Huh7.5-GFPu cells with C7 at MOI of 5. At 1 d post post infection, we treated the cells with 20 μM MG132 for 12 h and then analysed the STAT2 and GFPu expression levels. Under this condition, MG132 treatment only partially restored STAT2 and GFPu expression (Supplementary Figure 5b).

**Figure 5.**
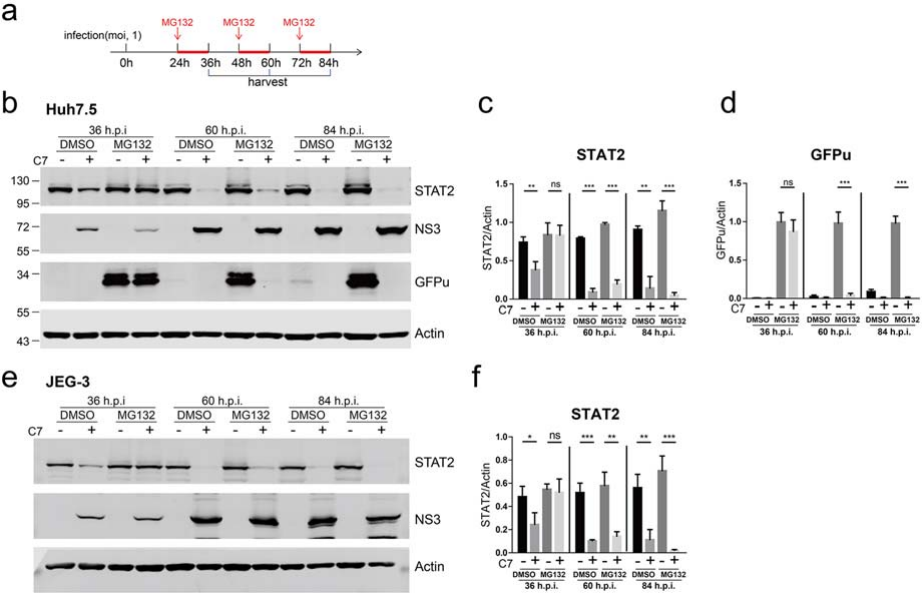
The proteasome inhibitor MG132 restored ZIKV infection-induced STAT2 degradation in a time-dependent manner. (a) The schematic of the experimental design for b–e. Huh7.5-GFPu (b-d) or JEG-3 cells (e-f) were infected with ZIKV C7 (MOI = 1). At the indicated time points post infection, the infected cells were treated with 10 μM DMSO or MG132 for 12 h and then harvested for western blotting with the indicated antibodies. (b) Representative pictures of Huh7.5-GFPu cells of three biological replicates are shown. The values to the left of the blots are molecular sizes in kilodaltons. (c–d) The protein abundances of each protein in B were quantified and plotted. The mean ± SD of three biological replicates is shown (*n* = 3). Statistical analysis was performed between C7-infected groups and uninfected groups (ns, not significant, *P < 0.05, **P < 0.01, ***P < 0.001; two-tailed, unpaired t-test). (e) Representative pictures of JEG-3 cells of three biological replicates are shown. The values to the left of the blots are molecular sizes in kilodaltons. (f) The protein abundances of each protein in e were quantified and plotted. The mean ± SD of three biological replicates is shown (*n* = 3). Statistical analysis was performed between C7-infected groups and uninfected groups (ns, not significant, *P < 0.05, **P < 0.01, ***P < 0.001; two-tailed, unpaired t-test).

We then carefully examined the restoration of STAT2 and GFPu expression by MG132 in ZIKV-infected cells. We infected Huh7.5-GFPu cells with C7 at an MOI of 1, treated the cells with 10 μM MG132 for 12 h at 1, 2 and 3 d post infection and then analysed the STAT2 and GFPu expression levels. When MG132 was added at 1 d post infection, it restored STAT2 and GFPu expression in the infected cells but did not restore STAT2 and GFPu expression at 2 d or 3 d post infection (Figure 5(a–d)). In addition, we repeated this experiment in human choriocarcinoma JEG-3 cell line and the results were consistent with those in Huh7.5 cells (Figure 5(e,f)). These data indicate that the proteasome inhibitor MG132 only restores ZIKV infection-induced STAT2 degradation at early time points of infection.

### ZIKV infection suppressed host *de novo* translation

**Figure 6.**
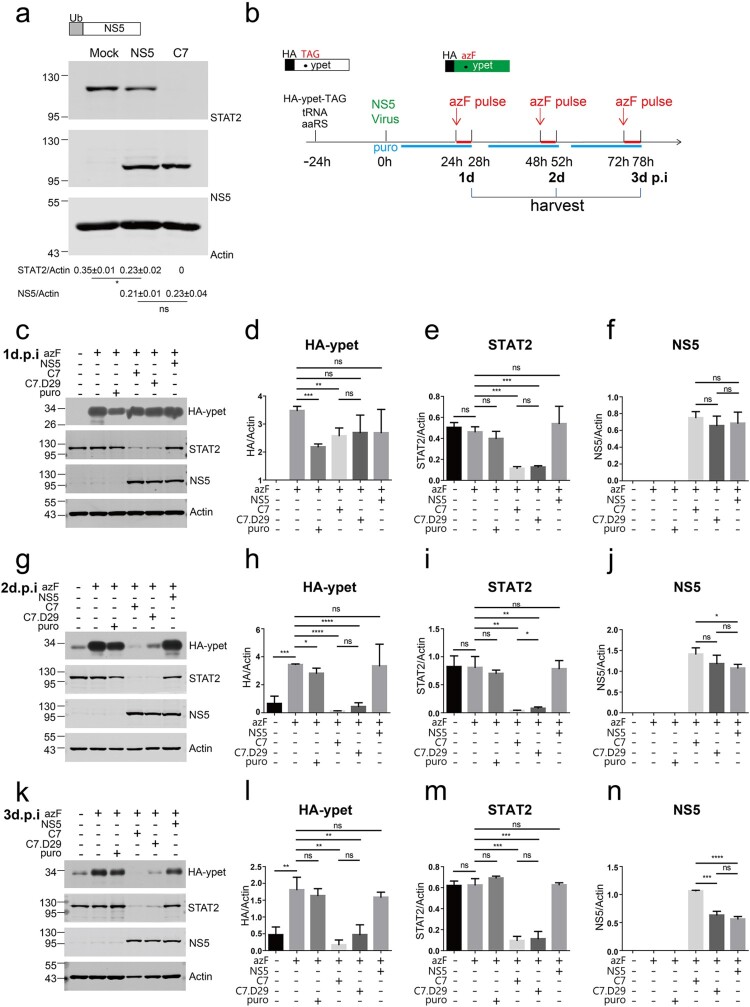
ZIKV infection interrupted host *de novo* translation. (a) Vero cells in duplicate wells were transfected with ZIKV Ubi-NS5-expressing plasmid (upper panel) or infected with ZIKV C7 (MOI = 1) for 54 h and then analysed by western blotting with the indicated antibodies. The protein abundances of each protein were quantified and plotted. A representative picture of two biological replicates is shown. Similar results were observed in multiple independent experiments. The values to the left of the blots are molecular sizes in kilodaltons. Statistical analysis was performed between the C7-infected group and the NS5-expressing group and the uninfected groups (Mock) (ns, not significant, *P < 0.05; two-tailed, unpaired *t*-test). (b) Schematic of the experimental design for c-n. The plasmids expressing the orthogonal tRNA (tRNA) and orthogonal aminoacyl-tRNA synthases (aaRS) and plasmid HA.ypet-TAG expressing the N-terminally HA-tagged ypet with the TAG codon replaced at amino acid 182 were cotransfected into Vero cells in triplicate wells for 24 h. Then, the transfected cells were infected or mock-infected (mock) with C7 (MOI = 5) or C7.D29 (MOI = 5) or retransfected with NS5. The cells were treated or not (control) with media containing p-azido-L-phenylalanine (0.5 mM) at 1, 2 and 3 d post infection and chased or not (control) for 4 h before harvest. For translation control, the cells were treated with 5 μg/ml puromycin (puro) for 24 h before harvesting at each time point. (c) Western blotting analysis of the 24-hour (1d)-infected cell lysates with the indicated antibodies. Representative pictures of three biological replicates are shown. The values to the left of the blots are molecular sizes in kilodaltons. (d–f) The protein abundances of protein bands in c were quantified and plotted. The mean ± SD of three biological replicates is shown (*n* = 3). Statistical analysis was performed between the indicated pairs (ns, not significant, *P < 0.05, **P < 0.01, ***P < 0.001; two-tailed, unpaired *t*-test). (g) Western blotting analysis of the 48-hour (2d)-infected cell lysates with the indicated antibodies. Representative pictures of three biological replicates are shown. The values to the left of the blots in the panels are molecular sizes in kilodaltons. (h–j) The protein abundances of each protein in g were quantified and plotted. The mean ± SD of three biological replicates is shown (*n* = 3). Statistical analysis was performed between the indicated pairs (ns, not significant, *P < 0.05, **P < 0.01, ***P < 0.001; two-tailed, unpaired *t*-test). (k) Western blotting analysis of the 72-hour (3d)-infected cell lysates with the indicated antibodies. Representative pictures of three biological replicates are shown. The values to the left of the blots are molecular sizes in kilodaltons. (l–n) The protein abundances of each protein in k were quantified and plotted. The mean ± SD of three biological replicates is shown (*n* = 3). Statistical analysis was performed between the indicated pairs (ns, not significant, *P < 0.05, **P < 0.01, ***P < 0.001; two-tailed, unpaired *t*-test).

Previous studies have reported that in cooperation with UBR4, DENV NS5 binds to STAT2 and degrades STAT2 in a proteasome-dependent manner [24,45]. As ZIKV infection induces degradation of STAT2 in a proteasome-dependent manner [22,23], we examined whether ZIKV NS5 triggers proteasomal degradation. Ubi-ZIKV NS5 was overexpressed in Vero cells, and the N-terminal ubiquitin of NS5 was cleaved by cellular ubiquitin carboxy-terminal hydrolase to produce the authentic N-terminal residues of NS5. Compared with C7 infection, overexpression of NS5 only marginally reduced STAT2 expression. Similar expression levels of NS5 were observed in the NS5-overexpressing cells and in the C7-infected cells (Figure 6(a)), suggesting the existence of new antagonism mechanisms. Given that ZIKV infection accelerated proteasomal degradation of ubiquitinated proteins and that the proteasome inhibitor MG132 only restored degradation at early time points of viral infection, we suspected that ZIKV infection interrupted the *de novo* synthesis of host ubiquitinated proteins.

It has been reported that dengue virus (DENV) and ZIKV lead to potent repression of host cell translation initiation, while the synthesis of viral proteins remains unaffected [46]. We adopted a bioorthogonal system to monitor the synthesis of new host proteins, in which amber suppressor tRNA/aminoacyl-tRNA synthetase (aaRS) pairs are orthogonal to mammalian tRNAs and synthetases. Unnatural amino acids (UAAs) can be recognized by aminoacyl-tRNA synthetase and introduced by amber suppressor tRNA orthogonal to the amber codon (UAG) during protein translation. Pulse incorporation of UAA into the translated protein indicates the efficiency of protein translation (Supplementary Figure 6). We employed an orthogonal suppressor tRNA/aminoacyl-tRNA synthetase (aaRS) pair [47], which could incorporate p-azido-L-phenylalanine (azF) into the amber codon (Figure 6(b)). We used an N-terminally HA-tagged ypet with amino acid 182 replaced with an amber codon. When the plasmids expressing the orthogonal tRNA (tRNA), orthogonal aminoacyl-tRNA synthases (aaRS) and HA.ypet-TAG were cotransfected into Vero cells, and in the presence of azF, translation ensued to produce full-length ypet, as evidenced by fluorescence microscopy. In contrast, in the absence of azF, no obvious fluorescence signal was detected (Supplementary Figure 7). We then infected the transfected cells with ZIKV C7 or C7.D29 at an MOI of 5 or retransfected the cells with the ZIKV Ub-NS5 plasmid. At 1, 2 and 3 d post infection, we treated the cells with azF for 4 h and then monitored the incorporation of azF by monitoring HA-ypet expression and observing ypet fluorescence. For protein translation inhibition control, we treated the cells with 5 μg/ml puromycin, which inhibits protein synthesis by disrupting peptide transfer on ribosomes [48]. As expected, at 1 d post infection, puromycin treatment significantly reduced HA-ypet expression. Likewise, infection with C7 but not C7.D29 significantly reduced HA-ypet expression (Figure 6(c,d)), suggesting a reduction in new protein synthesis. At 2 d and 3 d post infection, both C7 and C7.D29 infection dramatically and significantly reduced HA-ypet expression (Figure 6(g,h,k,l) and Supplementary Figure 7). In contrast, the expression of Ubi-NS5 did not affect HA-ypet expression, although the NS5 expression level was comparable to those in C7 and C7.D29-infected cells (Figure 6(c,d,f,g,h,j,k,l,n)). These data indicate that ZIKV infection but not NS5 expression interrupts host *de novo* translation. Notably, there was residual leaked HA-ypet expression in the absence of azF (Figure 6(g,k)), and the infected cells became insensitive to puromycin at 2 d and 3 d post infection, as puromycin did not efficiently block translation (Figure 6(g,h,k,l)), which was probably due to the overgrowth of the cells. Additionally, we repeated these experiments in glioblastoma SF268 cell line and the results were consistent with those in Vero cells (Supplementary Figure 8).

To generalize the concept to other flaviviruses, we repeated the experiment with YFV 17D. Similar to ZIKV, YFV infection significantly reduced HA-ypet expression (Supplementary Figure 9b, 9c, 9e) at 1 d and 2 d post infection.

### ZIKV infection did not affect proteasome activity *per se*

To examine whether ZIKV affects the activity of the proteasome *per se*, we need to monitor the proteasome activity of ZIKV-infected cells. First, we set up an experimental system to detect proteasome activity in Huh7.5-GFPu cells. As described before [40,49,50], cell lysates were incubated with a synthetic fluorogenic substrate (Suc-Leu-Leu-Val-Tyr-AMC) for proteasome analysis. Upon proteasome-mediated degradation, the fluorogenic substrate gives a fluorescent signal that indicates proteasome activity. As expected, treatment with the proteasome inhibitor MG132 dramatically reduced the fluorescence signal (Supplementary Figure 10a). Then, we monitored the proteasome activity in ZIKV-infected cells. We infected Huh7.5-GFPu cells with ZIKV C7 and then harvested the cell lysates at 1 d and 2 d post infection (Supplementary Figure 10b). We incubated the cell lysates with the fluorescently labelled substrate at 37°C for 90 min and then monitored the proteasome activity by determining the fluorescence intensity. ZIKV infection did not significantly affect host proteasome activity (Supplementary Figure 10c), suggesting that ZIKV had no effect on proteasome activity *per se*.

### Murine STAT2, which was refractory to ZIKV-induced ablation, elicited robust antiviral signalling upon IFN treatment

**Figure 7.**
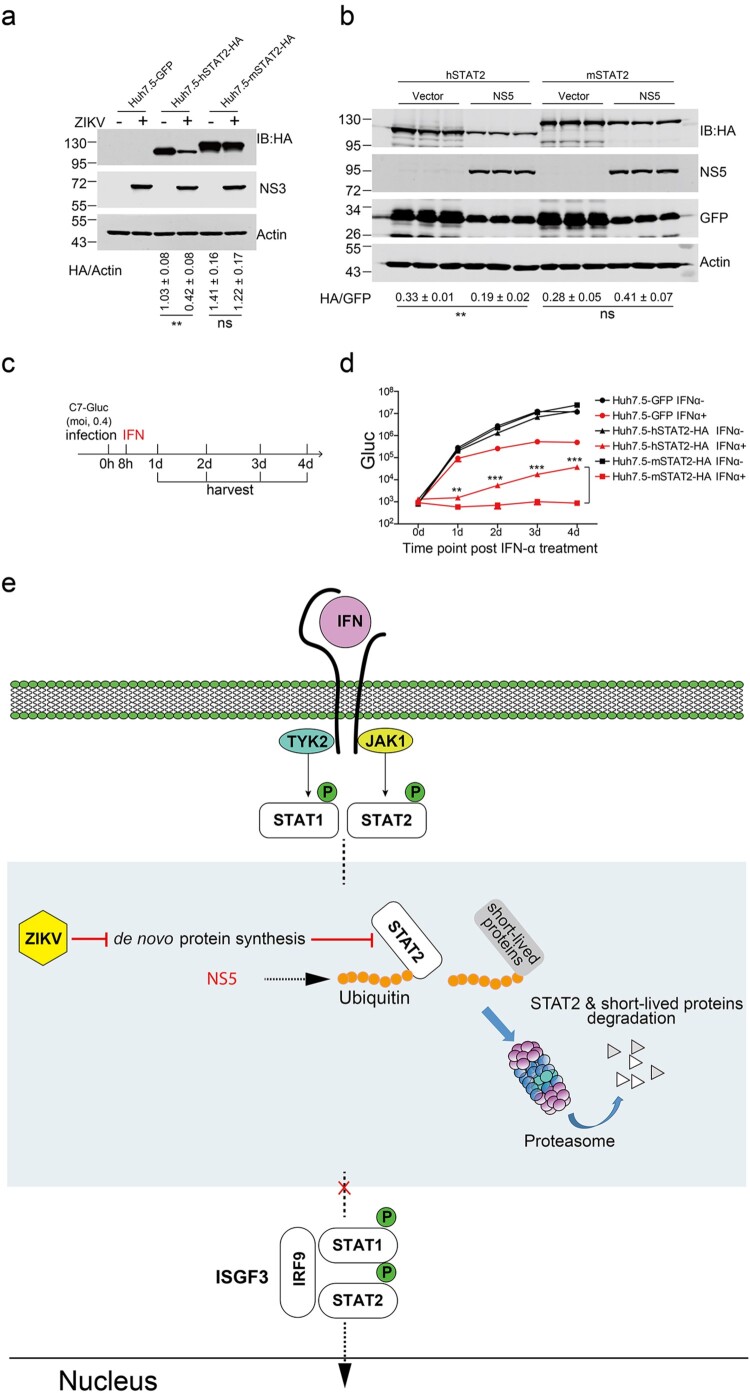
Murine STAT2, which was refractory to ZIKV-induced ablation, elicited robust antiviral signalling upon IFN treatment. (a) Murine STAT2 was refractory to ZIKV-induced reduction. Huh7.5 cells stably expressing GFP, hSTAT2-HA and murine STAT2-HA (mSTAT2-HA) were infected with ZIKV (MOI = 5) for 2 d and then harvested for western blotting analysis with the indicated antibodies. Representative pictures of three biological replicates are shown. The values to the left of the blots are molecular sizes in kilodaltons. Statistical analysis was performed between the C7-infected group and NS5-expressing group and the uninfected groups (Mock) (ns, not significant, **P < 0.01; two-tailed, unpaired t-test). (b) Murine STAT2 was refractory to ZIKV NS5 induced reduction. The plasmids expressing the ZIKV Ubi-NS5 or Vector were co-transfected with hSTAT2-HA or murine STAT2-HA (mSTAT2-HA)-expressing plasmid and GFP-expressing plasmid (6:3:1) in to Vero cells for 48 h, and then harvested for western blotting analysis with the indicated antibodies. Representative pictures of three biological replicates are shown. The values to the left of the blots are molecular sizes in kilodaltons. Statistical analysis was performed between the indicated groups (ns, not significant, **P < 0.01; two-tailed, unpaired t-test). (c) Experimental design for C. Huh7.5-GFP, Huh7.5-hSTAT2-HA and Huh7.5-mSTAT2-HA cells were infected with C7-Gluc (MOI = 0.4) for 8 h and then treated with IFN-α (2000 U/ml). At the indicated time points after IFN-α treatment, cells were harvested. (d) Gluc activities in the cell lysates of Huh7.5-GFP, Huh7.5-hSTAT2-HA and Huh7.5-mSTAT2-HA cells were determined. The mean ± SD of three biological replicates is shown (*n* = 3). Statistical analysis was performed between the indicated groups at each time point (**P < 0.01, ***P < 0.001; two-tailed, unpaired t-test). (e) Type I interferon (IFN) binds to interferon receptors, activating the Janus kinases Jak1 and Tyk2 to engage STAT1 and STAT1 and phosphorylate STAT1 and STAT2. Phosphorylated STAT1 and STAT2 form a heterodimer and recruit IFN regulatory factor 9 (IRF-9) to assemble interferon-stimulating gene factor 3 (ISGF3). ISGF3 enters the nucleus to elicit the expression of interferon-stimulating genes. ZIKV infection interrupts host de novo protein synthesis, accelerating the degradation of the pool of ubiquitinated short-lived proteins and STAT2. As reported, NS5 may contribute to this process by facilitating NS5 ubiquitination. See details in the text.

Previous studies have shown that ZIKV and DENV NS5 bind and degrade hSTAT2 but not murine STAT2 (mSTAT2), which may contribute to host tropism [22,51]. We constructed lentiviral plasmids expressing murine STAT2 and its human counterparts with an HA tag on the C termini as described above and then generated stable Huh7.5 cell lines expressing mSTAT2. Upon ZIKV infection at an MOI of 5, hSTAT2 was readily degraded, as evidenced by a reduction in the protein levels of STAT2-HA, whereas the protein levels of mSTAT2 were not affected (Figure 7(a)).

In order to detect the effect of ZIKV NS5 on the expression of human STAT2 and mouse STAT2, the plasmids expressing the ZIKV Ubi-NS5 or Vector were co-transfected with hSTAT2-HA or murine STAT2-HA (mSTAT2-HA)-expressing plasmid and GFP-expressing plasmid into Vero cells for 48 h, and then harvested for western blotting analysis. The results showed that expression of ZIKV NS5 decreased the protein level of human STAT2 but not that of mouse STAT2 (Figure 7(b)).

To explore the physiological relevance of the resistance of mSTAT2 to ZIKV-induced degradation, we infected Huh7.5-mSTAT2 cells with C7-Gluc at MOI of 0.4. Then, we treated the cells with or without IFN and assessed viral replication by measuring Gluc activity (Figure 7(c)). In the absence of IFN, hSTAT2 and mSTAT2 did not exhibit antiviral activity. Upon IFN-α treatment, in Huh7.5-hSTAT2 cells, exogenous hSTAT2 dramatically augmented IFN signalling, resulting in an approximately 3-log reduction in Gluc activity, and the antiviral effect was diminished at late time points post infection (Figure 7(d)), probably due to the virus-induced degradation of hSTAT2-HA. Strikingly, in Huh7.5-mSTAT2 cells, upon IFN-α treatment, mSTAT2 exhibited stronger antiviral activity than that of hSTAT2, and viral replication was almost totally inhibited (Figure 7(d)).

### Discussion

Previous studies have reported that ZIKV exploits various mechanisms to antagonize type I IFN signalling. ZIKV NS5 mediates the degradation of human STAT2 in a proteasome-dependent manner [22,23], but the effect of ZIKV on the ubiquitin proteasome is not clear, and there is no direct evidence that ZIKV reduces the expression of ubiquitinated STAT2.

In this study, consistent with previous reports, we observed a reduction in STAT2 in ZIKV-infected cells (Supplementary Figure 3a, Figure 6(a)), but overexpression of ZIKV NS5 alone did not reduce STAT2 to a similar level (Figure 6(a)). By using a ubiquitin proteasome reporter system, we found that ZIKV infection accelerated proteasomal degradation of host ubiquitinated proteins (Figure 3). However, ZIKV infection did not affect proteasome activity *per se* (Supplementary Figure 10). Mechanistically, by using a biorthogonal system, we demonstrated that ZIKV infection suppresses host *de novo* protein synthesis (Figure 6 and supplementary Figure 8). The observed accelerated degradation of ubiquitinated proteins was probably due to the suppression of *de novo* protein synthesis (Figure 7(e)). Recently, it has been reported that suppression of host *de novo* protein synthesis is a hallmark of flavivirus infection [46,52,53]. We propose that it might be a common strategy for flaviviruses to suppress host *de novo* protein synthesis to accelerate proteasomal degradation of short-lived antiviral proteins to antagonize IFN signalling, as YFV similarly suppressed host *de novo* protein synthesis (Supplementary Figure 9).

In this study, using biorthogonal system, we found that ZIKV infection suppressed host *de novo* translation, and reduce the in the expression of STAT2, while ZIKV NS5 did not suppressed host de novo translation. In line with previous reports [22,23,54,55], we found that exogenous murine STAT2 was refractory to ZIKV infection-induced degradation (Figure 7(a)), which is probably due to the failure of NS5 recognition of murine STAT2. Supporting this point, we found that overexpression of ZIKV NS5 did not affect murine STAT2 protein level (Figure 7(b)). We propose that ZIKV infection induces STAT2 ablation, probably through both the suppression of host *de novo* protein synthesis and the reported NS5-mediated degradation (Figure 7(e)).

ZIKV C7 antagonized interferon signalling at a high multiple of infection (MOI) and was sensitive to interferon signalling at a low MOI (Figures 1 and 2). This effect might be due to the accumulation of more viral antagonists, such as NS5, at high MOIs. Given that the expression of NS5 alone did not reduce STAT2 as ZIKV infection did (Figure 6(a)) and C7.D29 that exhibited less antagonist activity had similar viral protein levels as C7 (Figure 2(d)), it suggests other IFN antagonists exist in the ZIKV infected cells. It is possible that at high MOIs, ZIKV infection generates a high dosage of antagonists other than NS5 to suppress host *de novo* protein synthesis to ablate STAT2 signalling. Supporting this idea, ZIKV infection suppressed host *de novo* protein synthesis more dramatically at 2 d and 3 d post infection than at 1 d post infection (Figure 6(c,g,k)). C7.D29 is more sensitive to IFN than C7 and has slower replication kinetics (Supplementary Figure 1 and Figure 2d), which might also be due to slower kinetics of antagonizing signal generation.

In conclusion, we found that ZIKV employs novel IFN antagonism strategies by NS5-independent ablation of STAT2 to antagonize IFN signalling. Ectopic expression of murine STAT2 that was refractory to Zika virus-induced degradation elicited robust antiviral signalling. Further deciphering the molecular mechanisms of antagonizing signalling may provide novel insights into ZIKV immune evasion mechanisms and help in developing novel antiviral strategies.

## Supplementary Material

Supplementary_materials_TEMI-2021-0459-converted_editable.docx
