# Supplementary material for: NS5-independent Ablation of STAT2 by Zika virus to antagonize interferon signalling
Source: Emerg Microbes Infect. 2021 Aug 15;10(1):1609–25. doi: 10.1080/22221751.2021.1964384 (PMC8366623; doi:10.1080/22221751.2021.1964384)
Supplement: Supplementary_materials_TEMI-2021-0459-converted_editable.docx [file TEMI_A_1964384_SM0802.docx]

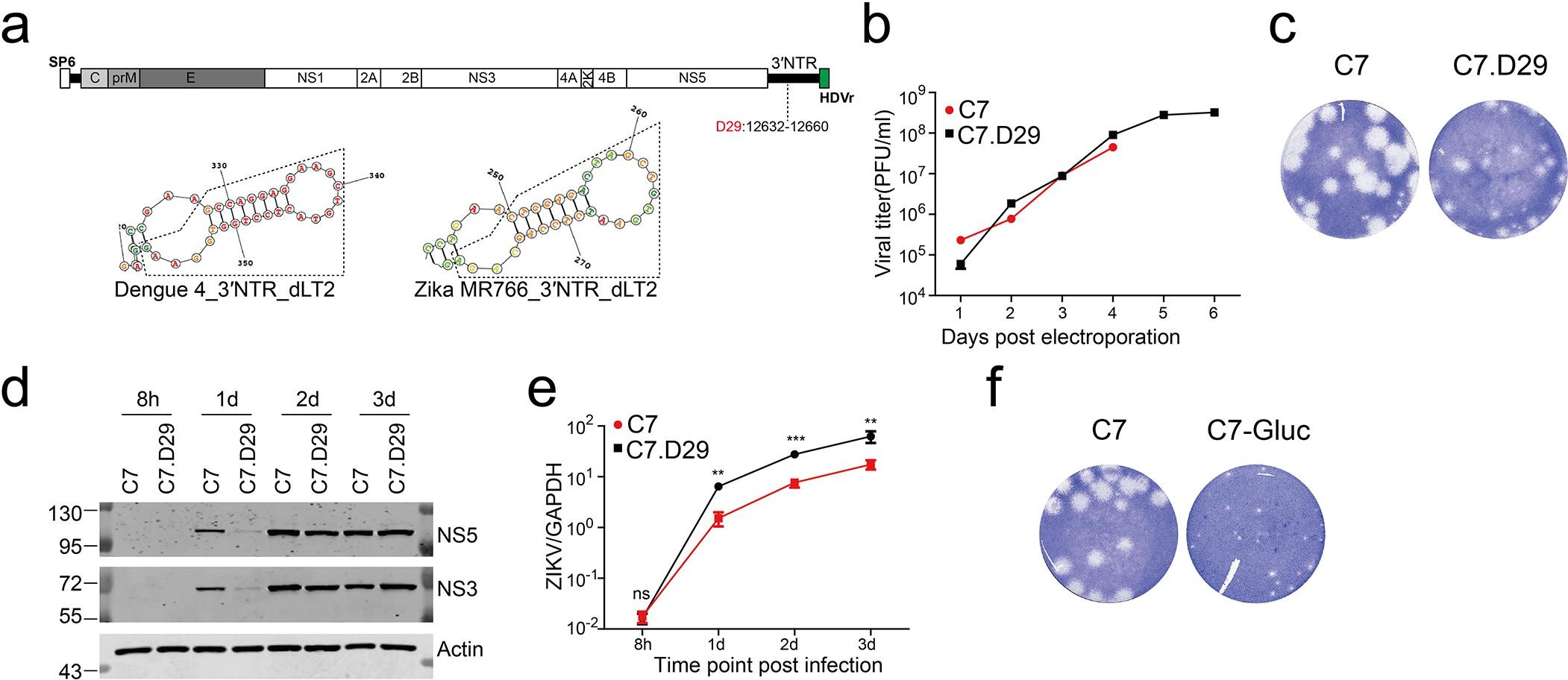


**Supplementary Figure 1. Characterization of infectious clone-derived Zika virus (ZIKV) and a ZIKV mutant.** (a) Schematic of pZikaMR766-C7.D29 (C7.D29) and the predicted stem loop in the 3'-nontranslated region (NTR) of dengue 4 virus and ZIKV MR766. The upper panel shows the schematic of pZikaMR766-C7.D29 (C7.D29). The lower panel shows the predicted stem loop in the 3'-nontranslated region (NTR) of dengue 4 virus and ZIKV MR766. The deletion regions are shown in the dashed boxes. (b) Growth curves of C7 and C7.D29. The in vitro-transcribed RNAs from pZikaMR766-C7 (C7) and pZikaMR766-C7.D29 (C7.D29) were transfected into Vero cells by electroporation. The cells were harvested on the indicated days post-electroporation (d.p.e.) and analyzed by plaque assays. (c) Representative plaque morphology of C7 (3 d.p.e.) and C7.D29 (3 d.p.e.) on Vero cells. (d) Huh7.5 cells were infected with C7 and C7.D29 at an MOI of 1 and then harvested at various time points post infection. Western blotting of the cell lysates with the indicated antibodies. A representative panel of three biological replicates is shown. The values to the left of the blots are molecular sizes in kilodaltons. (e) ZIKV RNA levels were quantified by quantitative

RT-PCR and normalized against GAPDH RNA levels. The mean ± SD of three biological

replicates is shown (n = 3). Statistical analysis was performed between C7 and C7.D29 (ns, not

significant, **P < 0.01, ***P < 0.001; two-tailed, unpaired t-test). (f) Representative plaque morphology of C7 (3 d.p.i.) and C7-Gluc (6 d.p.i.) on Vero cells.


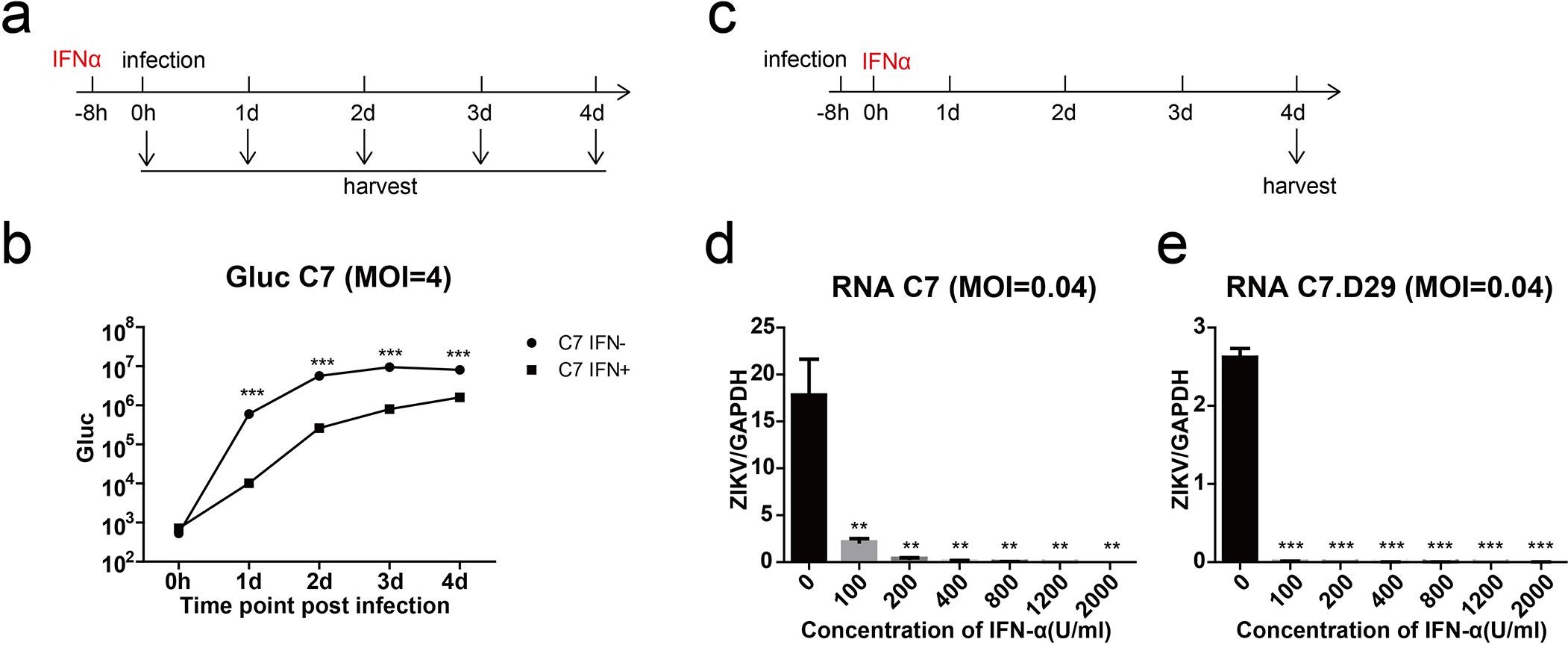


**Supplementary Figure 2. Effect of IFN on ZIKV infection.** (a) Schematic of Experimental design. Huh7.5 cells were treated with 2000 U/ml IFN-α and eight hours later, infected with C7-Gluc at an MOI of 4. Cells were harvested at the indicated time points post infection. (b) The Gluc activity were determined and plotted. The mean ± SD of three biological replicates is shown (n = 3). Statistical analysis was performed between the non-treated (IFN-) and the IFN-treated (IFN+) groups (***P

<0.001; two-tailed, unpaired *t*-test). (c) Schematic of Experimental design. Huh7.5 cells were infected with C7 or C7.D29 (MOI = 0.04) for 8 h and then treated with various concentrations of IFN-α. Cells were harvested at 4 d post infection. (d-e) ZIKV RNA from infected cells was

quantified by RT-PCR and normalized to the level of GAPDH RNA. The mean ± SD of three

biological replicates is shown (n = 3).


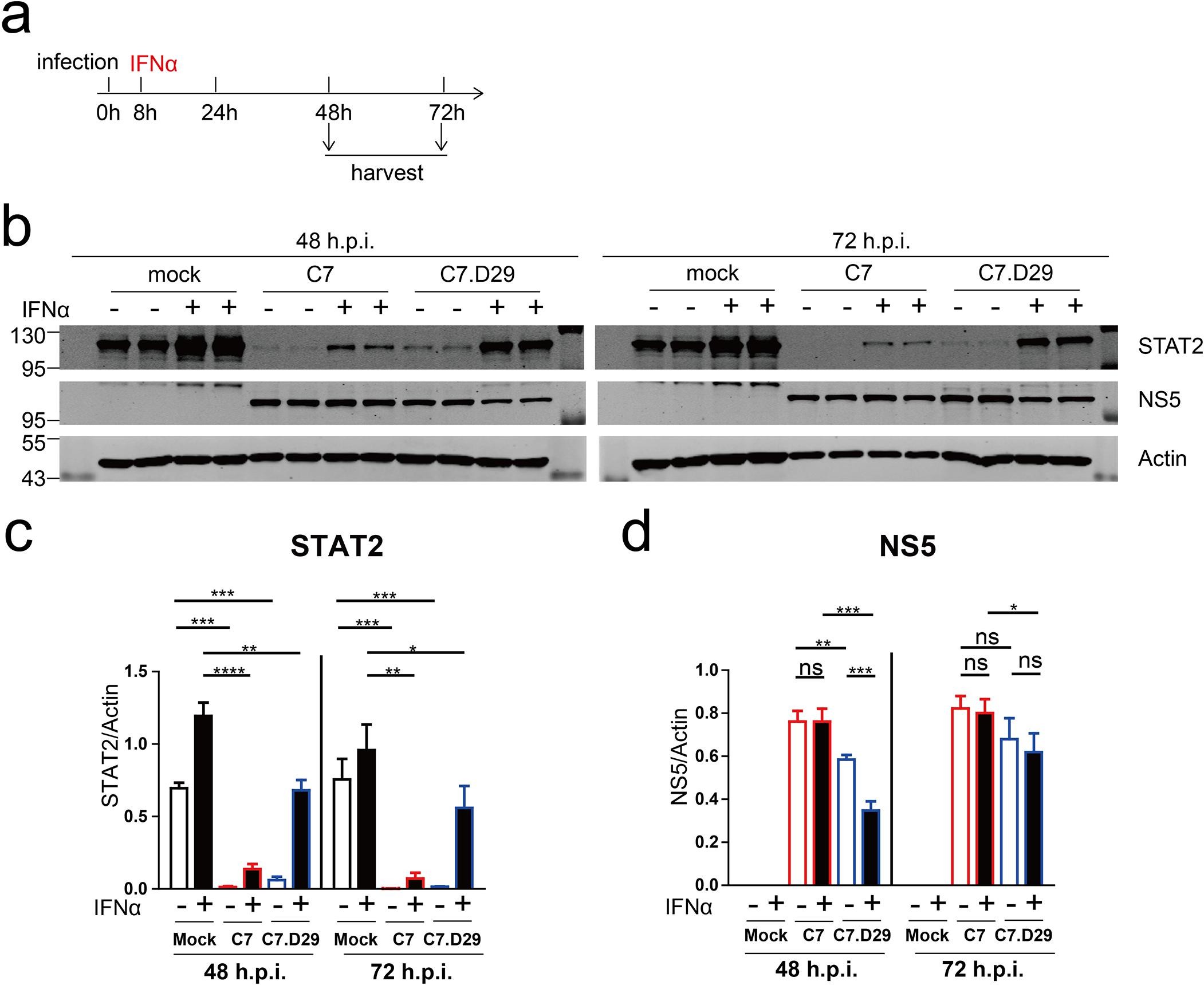


**Supplementary Figure 3. ZIKV infection induced a reduction in STAT2 protein levels.** (a) Schematic representation of the experimental design. Huh7.5 cells were infected with C7 (MOI = 5) or C7.D29 (MOI = 5) for 8 h and then treated with IFN-α (400 U/ml). Cells were harvested at 48 h and 72 h post infection. (b) The cells were analyzed by western blotting with indicated antibodies. A representative picture of three biological replicates is shown. The values to the left of the blots are molecular sizes in kilodaltons. (c-d) The protein abundances of each protein in b were quantified and plotted. The mean ± SD of three biological replicates is shown (n = 3). Statistical analysis was performed between the C7- or the C7.D29-infected groups and the uninfected groups (ns, not significant; *P < 0.05; **P < 0.01; ***P < 0.001; two-tailed, unpaired *t*-test).


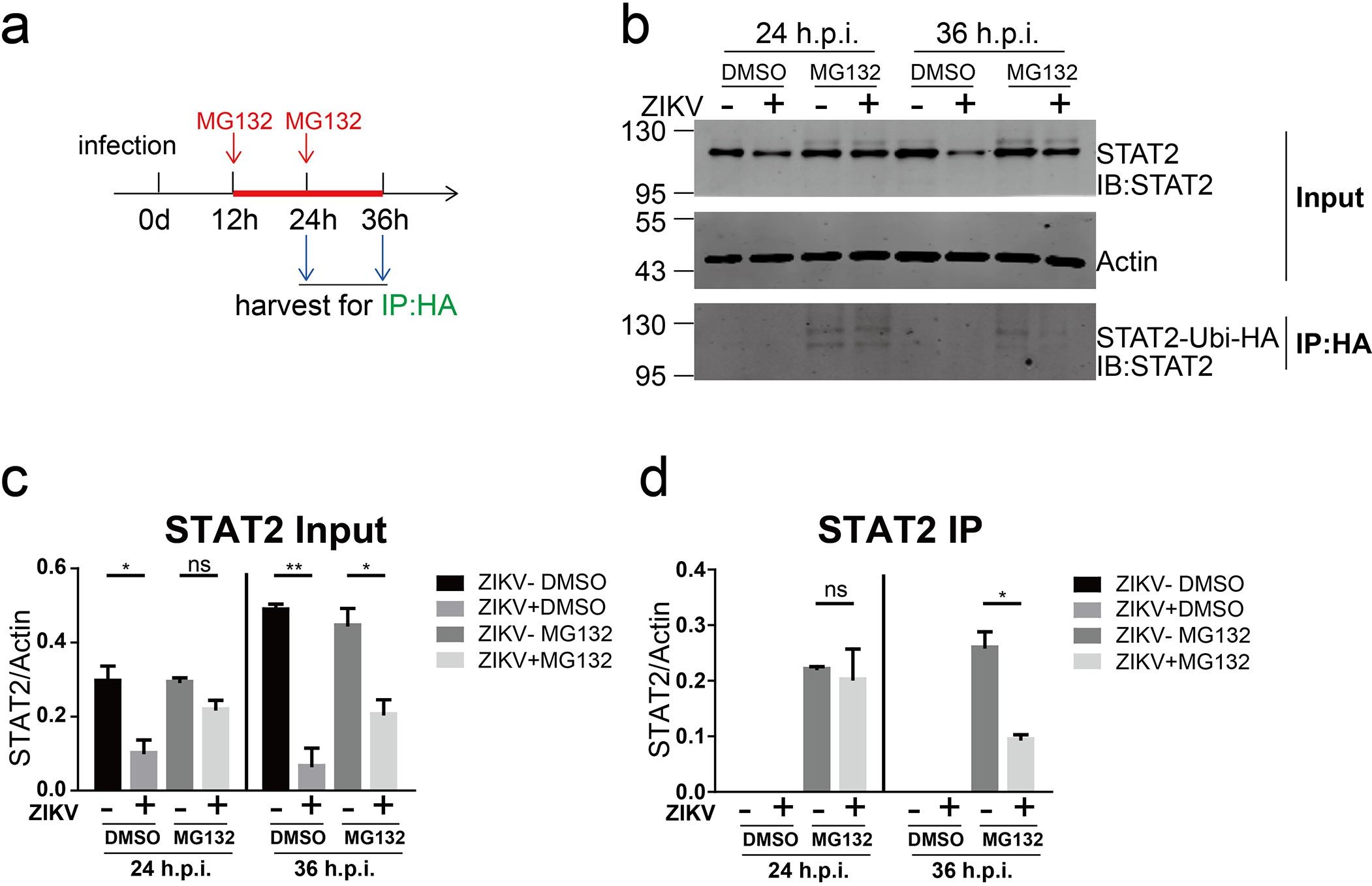


**Supplementary Figure 4. ZIKV infection induced degradation of ubiquitinated STAT2.** (a) The schematic of the experimental design for B-D. Huh7.5-HA-Ub cells were infected with ZIKV (MOI

= 1). At 12 h or 24 h hours post infection, the infected cells were treated with 10 μM of DMSO or MG132 for another 12 hours and then harvested for immunoprecipitation (IP) with anti-HA antibody.

(b) The cell lysates (Input) and the immunoprecipitated complex were analyzed by western blotting with indicated antibodies. A representative picture of three biological replicates is shown. The values to the left of the blots are molecular sizes in kilodaltons. (c-d) The protein abundances of each protein in B were quantified and plotted. The mean ± SD of three biological replicates is shown (n = 3). Statistical analysis was performed between the C7-infected groups and the uninfected groups (ns, not significant; *P < 0.05; **P < 0.01; two-tailed, unpaired *t*-test).


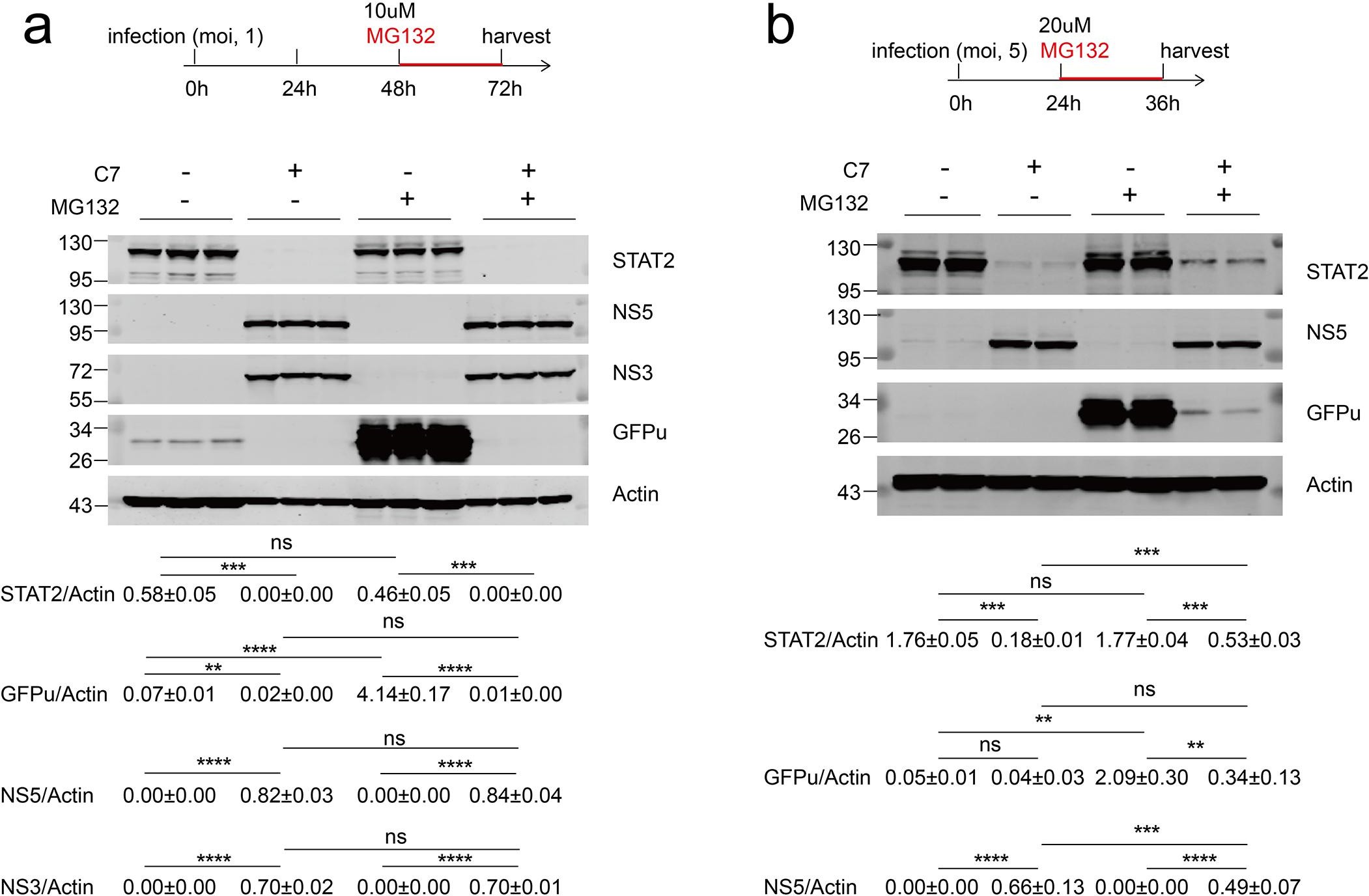


**Supplementary Figure 5. The proteasome inhibitor MG132 could not restored ZIKV infection-induced STAT2 degradation at late time point.** (a) The upper panel shows the schematic of the experimental design. Huh7.5-GFPu cells in triplicates were infected with ZIKV (MOI = 1) for 48 h and then treated with MG132 (10 μM) for 24 h and harvested. The cell lysates were analyzed by western blotting with indicated antibodies. The values to the left of the blots are molecular sizes in kilodaltons. The protein abundances of each protein were quantified and plotted. The mean ± SD of three biological replicates is shown (n = 3). Statistical analysis was performed between the indicated pairs (ns, not significant, *P < 0.05; **P < 0.01; ***P < 0.001; two-tailed, unpaired *t*-test). (b) The upper panel shows the schematic of the experimental design. Huh7.5-GFPu cells were infected with ZIKV (MOI = 5) for 24 h and then treated with MG132 (20 μM) for 12 h and harvested. The cell lysates were analyzed by western blotting with indicated antibodies. The values to the left of the blots are molecular sizes in kilodaltons. The protein abundances of protein bands were quantified and plotted. The mean ± SD of three biological replicates is shown (n = 3). Statistical analysis was performed between the indicated pairs (ns, not significant, *P<0.05; **P<0.01; ***P<0.001; two-tailed, unpaired *t*-test).


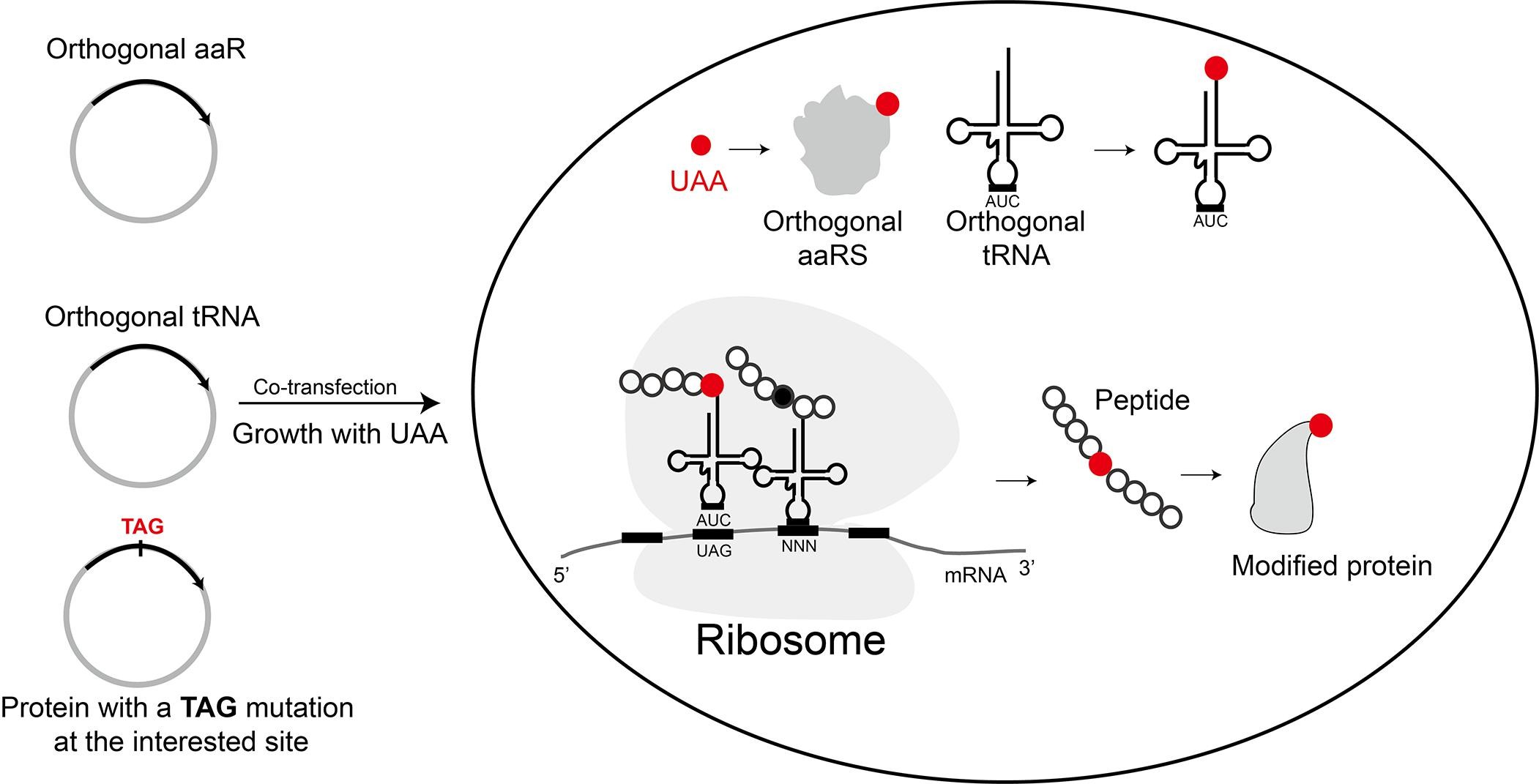


**Supplementary Figure 6. Schematic of bioorthogonal system.** Plasmids expressing the orthogonal tRNA (tRNA) and the paired orthogonal aminoacyl-tRNA synthases (aaRS) were co-transfected with the plasmid expressing protein of interest with amber codon (TAG) inserted in the open reading into cells. The amber suppressor tRNA/aminoacyl-tRNA synthetase (aaRS) pair is orthogonal to mammalian tRNAs and synthetases. In the presence of unnatural amino acids (UAAs), the UAA can be introduced by the orthogonal pair into the amber codon (UAG) during protein translation. Pulse incorporation of UAA into the translated protein indicates the efficiency of protein translation.


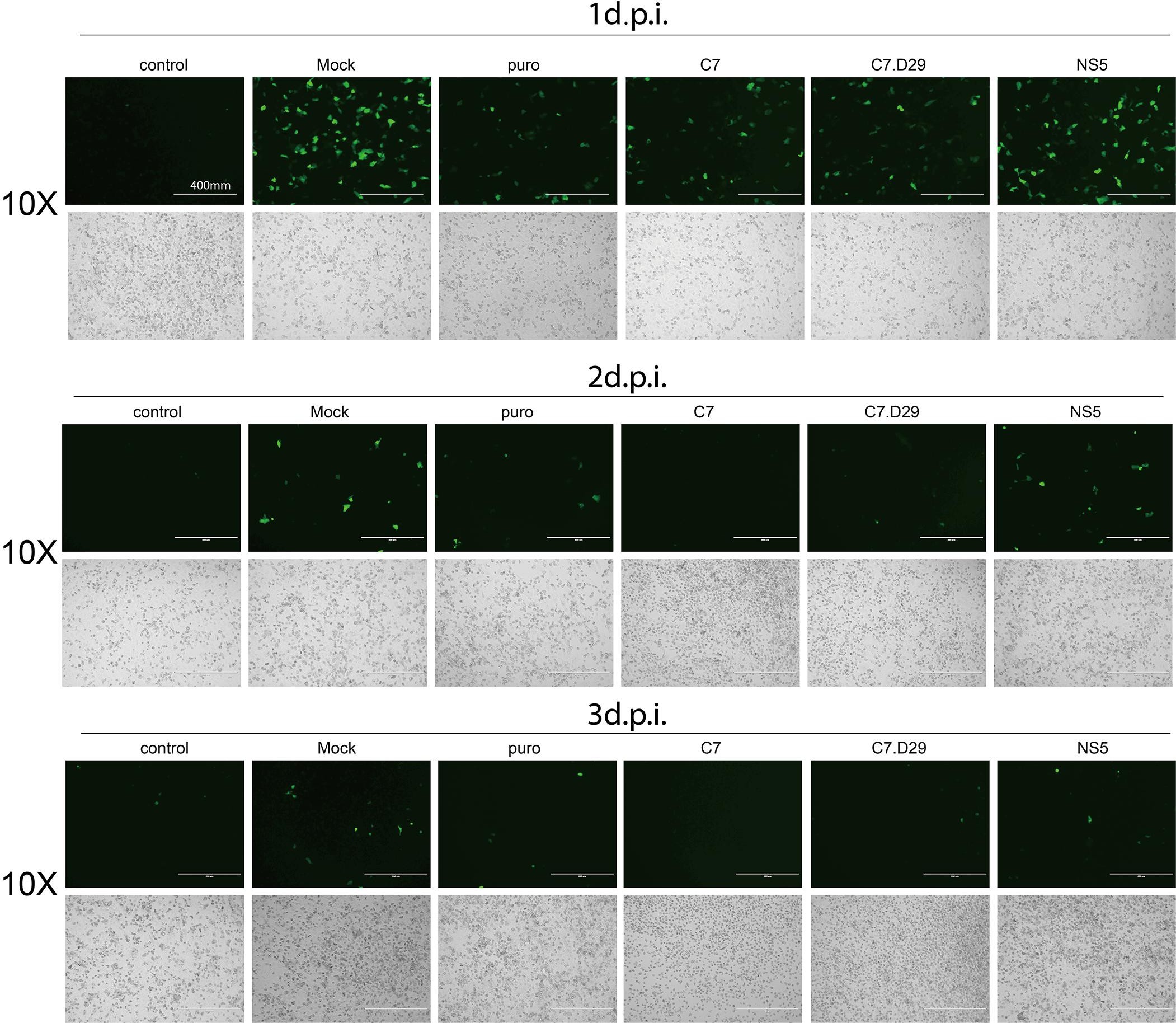


**Supplementary Figure 7. ZIKV infection suppressed host *de novo* translation.** Plasmids expressing the orthogonal tRNA (tRNA), orthogonal aminoacyl-tRNA synthases (aaRS) and plasmid HA.ypet-TAG expressing the N-terminally HA-tagged ypet with TAG codon replaced at amino acid 182 were cotransfected into Vero cells in triplicate wells. After 24 h, the transfected cells were infected with C7 (MOI = 5) or C7.D29 (MOI = 5) or retransfected with NS5. The cells were treated or not (control) with media containing p-azido-L-phenylalanine (0.5 mM) at 1 d, 2 d and 3 d post infection and chased or not (control) for 4 hours before harvest. For translation control, the cells were treated with 5 μg/ml puromycin (puro) for 24 hours before harvesting at each time point. At 1 d, 2 d and 3 d post infection, cells were treated or not (control) with media containing azF (0.5 mM) and then chased for 4 hours and observed by fluorescence microscopy. Scar bar, 400μm.


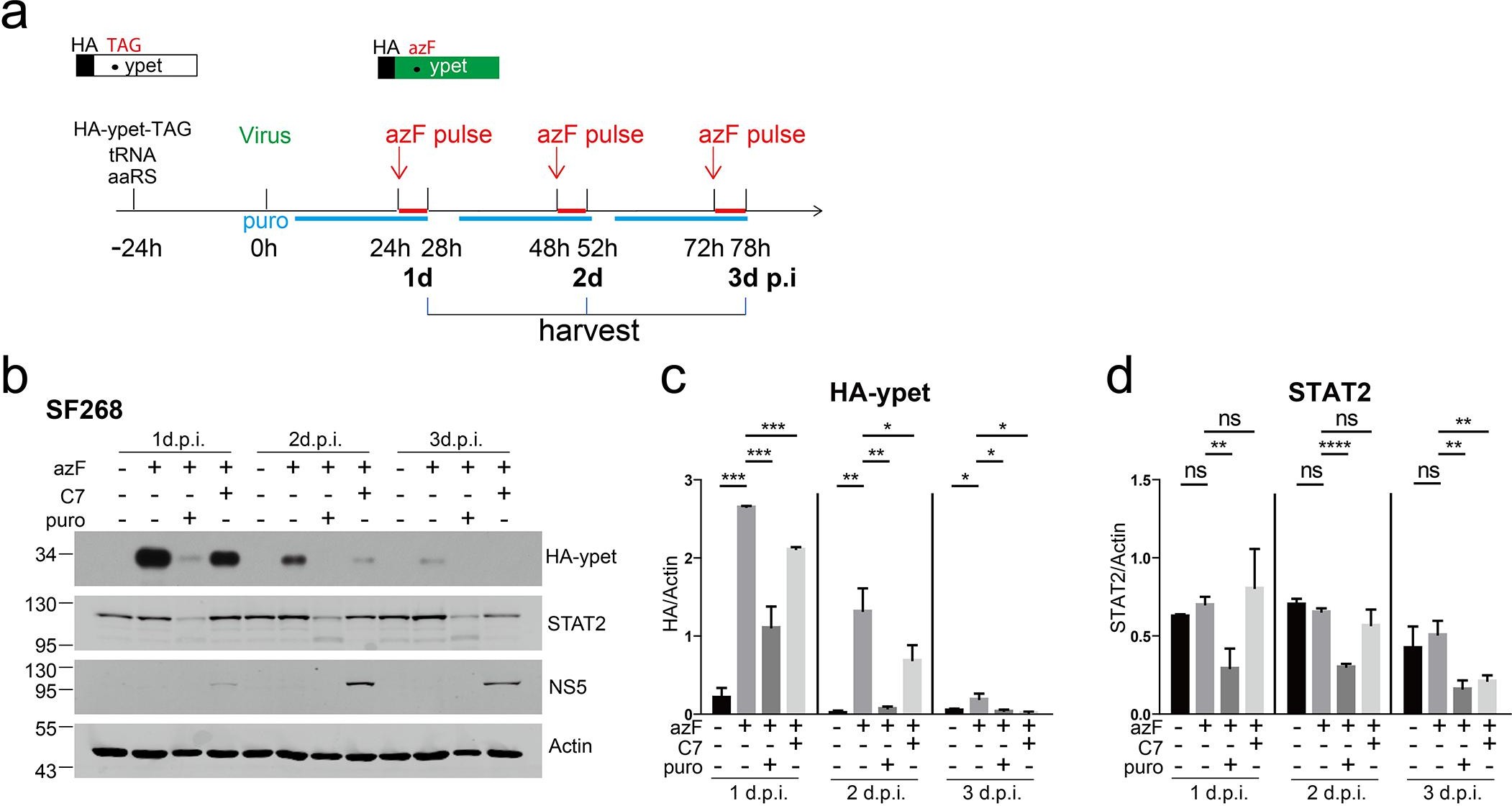


**Supplementary Figure 8. ZIKV infection interrupted host de novo translation in SF268 cell line.**

(a) Schematic of the experimental design for b-d. The plasmids expressing the orthogonal tRNA (tRNA) and orthogonal aminoacyl-tRNA synthases (aaRS) and plasmid HA.ypet-TAG expressing the N-terminally HA-tagged ypet with the TAG codon replaced at amino acid 182 were cotransfected into SF268 cells in triplicate wells for 24 h. Then, the transfected cells were infected or mock-infected (mock) with ZIKV (MOI = 10). The cells were treated or not (control) with media containing p-azido-L-phenylalanine (0.5 mM) at 1 d or 2 d post infection and chased or not (control) for 4 hours before harvest. For translation control, the cells were treated with 5 μg/ml puromycin (puro) for 24 hours before harvesting at each time point. (b) western blotting analysis of the 24-hour (1d) and 48-hour (2d) and 72-hour (3d)-infected cell lysates with the indicated antibodies. Representative pictures of three biological replicates are shown. The values to the left of the blots are molecular sizes in kilodaltons. (c-d) The protein abundances of protein bands in b were quantified and plotted. The mean ± SD of three biological replicates is shown (n = 3). Statistical analysis was performed between the indicated pairs (ns, not significant, *P < 0.05, **P < 0.01, ***P < 0.001; two-tailed, unpaired t-test).


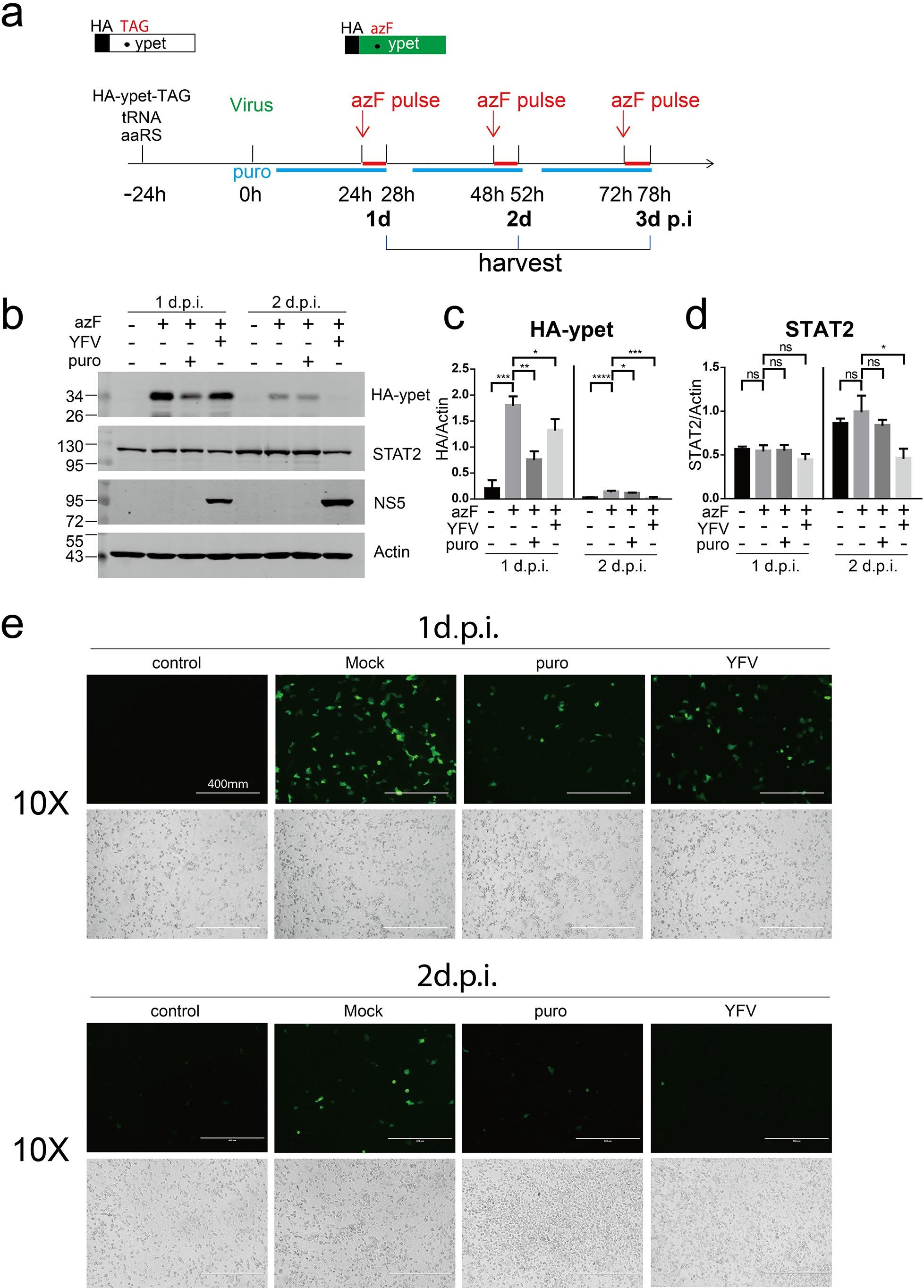


**Supplementary Figure 9. YFV infection interrupted host de novo translation.** (a) Schematic of the experimental design for B-E. The plasmids expressing the orthogonal tRNA (tRNA) and orthogonal aminoacyl-tRNA synthases (aaRS) and plasmid HA.ypet-TAG expressing the N-terminally HA-tagged ypet with the TAG codon replaced at amino acid 182 were cotransfected into Vero cells in triplicate wells for 24 h. Then, the transfected cells were infected or mock-infected (mock) with YFV-17D (MOI = 5). The cells were treated or not (control) with media containing p-azido-L-phenylalanine (0.5 mM) at 1 d or 2 d post infection and chased or not (control) for 4 hours before harvest. For translation control, the cells were treated with 5 μg/ml puromycin (puro) for 24 hours before harvesting at each time point. (b) western blotting analysis of the 24-hour (1d)- and 48-hour (2d)-infected cell lysates with the indicated antibodies. Representative pictures of three biological replicates are shown. The values to the left of the blots are molecular sizes in kilodaltons.

(c-d) The protein abundances of protein bands in B were quantified and plotted. The mean ± SD of

three biological replicates is shown (n = 3). Statistical analysis was performed between the indicated

pairs (ns, not significant, *P < 0.05, **P < 0.01, ***P < 0.001; two-tailed, unpaired t-test). (e) Fluorescence microscopy results of A. At 1 d, 2 d and 3 d post infection, cells were treated or not (control) with media containing azF (0.5 mM) and then chased for 4 hours and observed by fluorescence microscopy. Scar bar, 400μm.

# Supplementary Figure 10


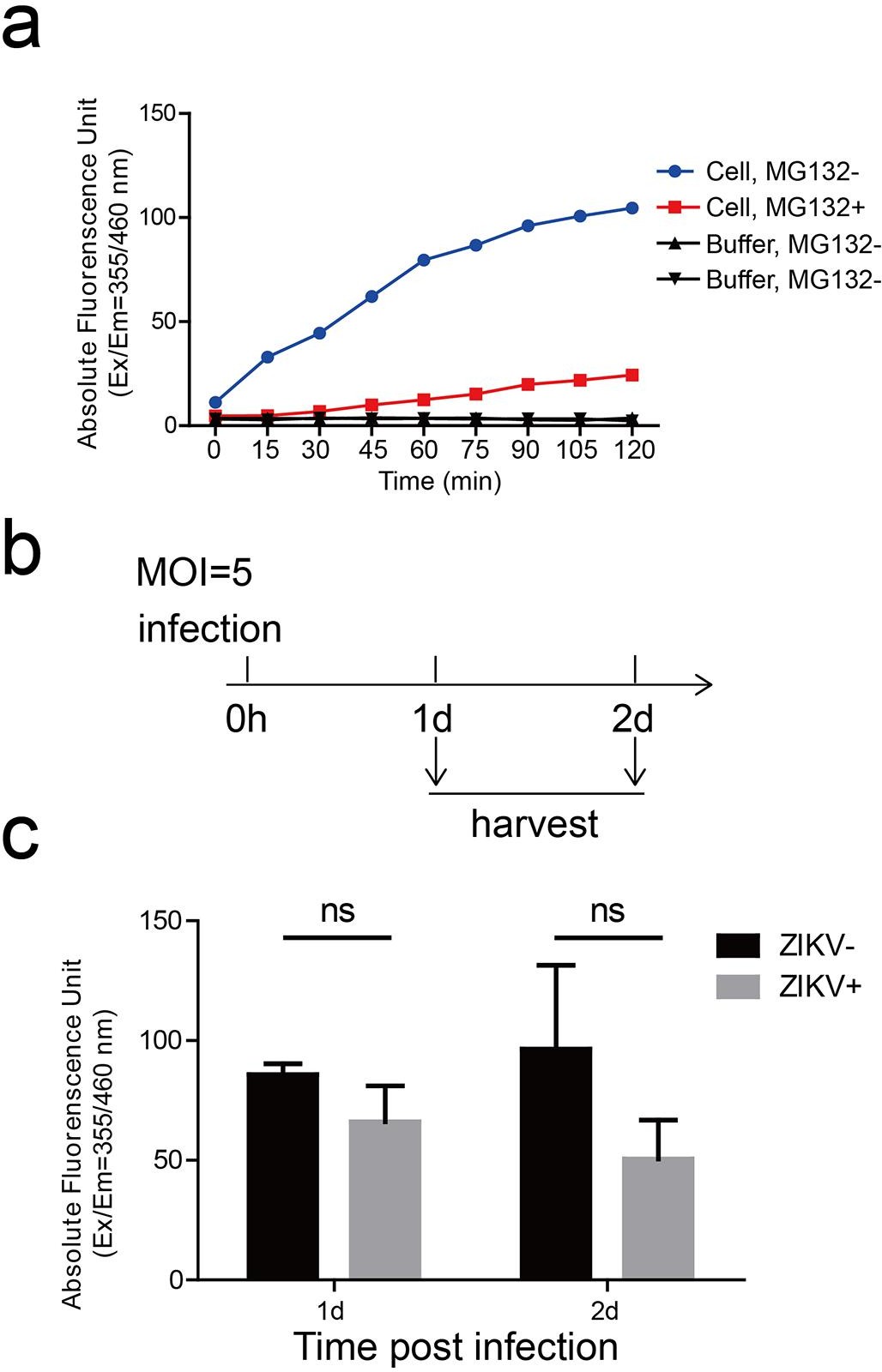


**Supplementary Figure 10. ZIKV infection did not affect proteasome activity *per se*.** (a) Proteasome activity assay of Huh7.5-GFPu cell. Ten microgram of cell lysates were incubated with the synthetic fluorogenic substrate (Suc-Leu-Leu-Val-Tyr-AMC) at 37°C and then the fluorescence intensity was monitored by a plate reader with excitation and emission filters of 355 nm and 460 nm, respectively. The values were counted per 15 min. (b) Experimental design for C. Huh7.5-GFPu cells were infected with ZIKV C7 (MOI = 5). At 1 d and 2 d post infection, cell lysates of the infected cells were incubated with the fluorescently labeled substrate at 37°C for 90 min and then the fluorescence intensity was monitored as described above. (c) Proteasome activity of cells in B were quantified and plotted. The mean ± SD of three biological replicates is shown (n = 3). Statistical analysis was performed between the C7-infected groups and the uninfected groups (ns, not significant; two-tailed, unpaired *t*-test).
